# Physical functional performance and prognosis in patients with heart failure: a systematic review and meta-analysis

**DOI:** 10.1186/s12872-020-01725-5

**Published:** 2020-12-09

**Authors:** Iván José Fuentes-Abolafio, Brendon Stubbs, Luis Miguel Pérez-Belmonte, María Rosa Bernal-López, Ricardo Gómez-Huelgas, Antonio Ignacio Cuesta-Vargas

**Affiliations:** 1 Department of Physiotherapy, Faculty of Health Science, University of Malaga, The Institute of Biomedical Research in Malaga (IBIMA), Clinimetric Group FE-14, Malaga, Spain; 2 Physiotherapy Department, South London and Maudsley NHS Foundation Trust, Denmark Hill, London, UK; 3 Department of Psychological Medicine, Institute of Psychiatry, Psychology and Neuroscience, King’s College London, London, UK; 4 Positive Ageing Research Intitute (PARI), Faculty of Health Social Care and Education, Anglia Ruskin University, Chelmsford, UK; 5 Internal Medicine Department, Instituto de Investigación Biomédica de Malaga (IBIMA), Regional University Hospital of Málaga, Málaga, Spain; 6 Unidad de Neurofisiología Cognitiva, Centro de Investigaciones Médico Sanitarias (CIMES), Instituto de Investigación Biomédica de Málaga (IBIMA), Universidad de Málaga (UMA), Campus de Excelencia Internacional (CEI) Andalucía Tech, Málaga, Spain; 7 Centro de Investigación Biomédica en Red Enfermedades Cardiovasculares (CIBERCV), Instituto de Salud Carlos III, Madrid, Spain; 8 CIBER Fisio-patología de la Obesidad y la Nutrición, Instituto de Salud Carlos III, Madrid, Spain; 9 School of Clinical Sciences, Faculty of Health at the Queensland University of Technology, Brisbane, Queensland Australia

**Keywords:** Functional tests, Heart failure, Hospitalisation, Mortality, Physical functional performance, Prognosis

## Abstract

**Background:**

Patients with Heart Failure (HF) show impaired functional capacities which have been related to their prognosis. Moreover, physical functional performance in functional tests has also been related to the prognosis in patients with HF. Thus, it would be useful to investigate how physical functional performance in functional tests could determine the prognosis in patients with HF, because HF is the leading cause of hospital admissions for people older than 65 years old. This systematic review and meta-analysis aims to summarise and synthesise the evidence published about the relationship between physical functional performance and prognosis in patients with HF, as well as assess the risk of bias of included studies and the level of evidence per outcome.

**Methods:**

Major electronic databases, such as PubMed, AMED, CINAHL, EMBASE, PEDro, Web of Science, were searched from inception to March 2020 for observational longitudinal cohort studies (prospective or retrospective) examining the relationship between physical functional performance and prognosis in patients with HF.

**Results:**

44 observational longitudinal cohort studies with a total of 22,598 patients with HF were included. 26 included studies reported a low risk of bias, and 17 included studies showed a moderate risk of bias. Patients with poor physical functional performance in the Six Minute Walking Test (6MWT), in the Short Physical Performance Battery (SPPB) and in the Gait Speed Test showed worse prognosis in terms of larger risk of hospitalisation or mortality than patients with good physical functional performance. However, there was a lack of homogeneity regarding which cut-off points should be used to stratify patients with poor physical functional performance from patients with good physical functional performance.

**Conclusion:**

The review includes a large number of studies which show a strong relationship between physical functional performance and prognosis in patients with HF. Most of the included studies reported a low risk of bias, and GRADE criteria showed a low and a moderate level of evidence per outcome.

## Background

Cardiovascular diseases continue to be the leading cause of disability-adjusted life-years (DALYs) due to non-communicable diseases and the leading cause of death [1–3]. Within cardiovascular diseases, Heart Failure (HF) is the only cardiovascular disease which is increasing in incidence and prevalence due to the aging of the world population, because its prevalence increases with age [4–8]. In addition, heart failure constitutes the most important hospital diagnosis in older adults, is the leading cause of hospital admissions for people older than 65 years old and contributes to the increase of medical care costs [5–9].

Heart Failure is characterised by a weak myocardium with decreased cardiac output that is unable to meet the body metabolic demands [4–6, 8, 10–12]. There are several functional symptoms that appear in patients with HF, such as reduced aerobic capacity, decreased muscle strength, low weekly physical activity and exercise intolerance, which are accompanied by fatigue and dyspnea symptoms [12–17]. Furthermore, patients with HF show impaired functional capacities, experience a declined ability to carry out their activities of daily living and suffer a reduced quality of life [12, 14, 17]. It has also been reported that patients with chronic HF show a slower gait speed than healthy subjects of the same age [18]. The maximal aerobic capacity has been inversely correlated to the severity of HF and has been directly correlated to the prognosis and the life expectancy [14, 19, 20]. Similarly, the lower extremities muscle mass and muscle strength have also been related to long-term survival in patients with HF [14, 21].

Some functional tests have been used to predict prognosis in patients with HF. Thus, the 6-min walk test (6-MWT) has been proposed as a simple, inexpensive, safe and reproducible exercise test to assess functional capacity in patients with HF, which could also predict the prognosis of patients with HF based on distance walked [12, 22–24]. The Short Physical Performance Battery (SPPB) provides a useful and indirect measure of muscle functional capacity [12]. Moreover, the SPPB and the Timed Up and Go test (TUG) could be used to assess physical or functional frailty in patients with HF, which has been associated with an increased risk of hospitalisation and mortality in chronic heart failure [25, 26]. The utility of Gait Speed ​​has also been shown to predict functional independence loss, cardiovascular disease, hospitalisation, and mortality in older adults [27–31]. The 6-MWT measures the distance which patients can walk during 6 min [32]. The test is usually conducted in a closed corridor of 30 m where two marks are placed on the ground at a distance of 30 m, and patients walk from one end to the other, during 6 min [32]. The SPPB includes 3 tests: balance (feet together, semitándem and tandem during 10 s each), gait speed (4 m) and standing up and sitting on a chair 5 times. Each test is scored from 0 (worst performance) to 4 (best performance). The total score for the whole battery that is the addition of the 3 tests and ranges from 0 to 12 [33]. In the TUG test patients are sat down in a chair, and at the order to “go”, they stand up from the chair, walk 3 m until a reach a line that is on the floor. Then, patients should turn, return to the chair walking and sit again [34].

Hence, it would be necessary to conduct a synthesis of evidence that explores the relationship between the physical functional performance in functional tests and the prognosis in patients with HF. A systematic review may permit the formation of firm conclusions through an exhaustive synthesis of data [35]. Thus, the aim of this study was to answer the following PECOS (P, participant; E, exposure; C, comparator; O, outcome; S, study design) question through a systematic review of the literature on observational longitudinal cohort studies (prospective or retrospective) (S): Do older patients with HF (P), who have poor physical functional performance in some functional tests, such as 6-MWT, SPPB, TUG or Gait Speed (E), show a worse prognosis (O) than those patients with good physical functional performance (C)?

## Methods

The Systematic Review and Meta-analysis was conducted in accordance with the Preferred Reporting Items for Systematic Reviews and Meta-Analyses (PRISMA) statement [36]. The systematic review protocol was registered at the International Prospective Register of Systematic Reviews (PROSPERO: CRD42020177427).

### Data sources and search strategy

Two independent reviewers (IJF-A and AIC-V) conducted a systematic search using relevant search terms that were developed from Medical Subject Headings (MeSH) and keywords from other similar studies from inception to March, 24th 2020 using optimised search strategies in the following electronic databases: PubMed, AMED, CINAHL, EMBASE, PEDro, Web of Science (Additional file 1). A manual search of relevant eligible studies, to select any studies missed during the electronic search, was also conducted using cross-references identified in the reference lists within both original and review articles. The grey literature databases, such as New York Academy of Medicine Grey Literature Report, Open Grey and Google Scholar [37] were examined to identify any relevant unpublished data. References were exported, and duplicates were removed using the Mendeley desktop V.1.19.2 citation management software.

### Eligibility criteria

The aforementioned PECOS framework was followed to determine which studies were included in the present systematic review and meta-analysis. Each study had to meet the following inclusion criteria:

1. Observational longitudinal cohort studies (prospective or retrospective)(S) examining whether older patients with HF (P), who have a poor physical functional performance in some functional tests, such as 6-MWT, SPPB, TUG or Gait Speed (E), show worse prognosis, assessed as larger risk of hospitalisation or mortality, (O) than those patients with good physical functional performance (C).
2. No restriction was applied on the participants’ age, ethnicity, gender, HF diagnosis or on the New York Heart Association (NYHA) scale score.
3. No restriction was applied on the language.
4. Studies recruiting participants from any setting (general population, primary or secondary care).
5. Studies providing Odds Ratio (OR) or Hazard Ratio (HR) data.

The exclusion criteria were as follows:

1. All studies that did not include an observational longitudinal cohort design (e.g cross-sectional studies, randomised controlled trials).
2. Studies exploring the prognosis value of functional tests in patients with other cardiovascular diseases different from HF.
3. Studies examining the relationship between physical functional performance in functional tests and other outcomes different from mortality or hospitalisation.
4. Studies investigating the prognosis value of physical activity assessed as daily activity, exercise time per week or physical activity scales.

### Study selection

Two independent reviewers (IJF-A and AIC-V) carried out the screening of titles and abstracts to detect potentially relevant records and also excluded those documents that were not original papers. The same reviewers conducted the screening of those articles that met all inclusion criteria. A short checklist was carried out and followed in order to select the relevant studies (Additional file 2). In case of disagreements, the articles were always included.

### Data extraction

Two independent reviewers (IJF-A and AIC-V) identified the following relevant data from each study: study details (first author and year of publication), region, setting, study design, sample size, functional tests with their cut-off points and characteristics of participants (mean age, %males), HF diagnosis, follow-up, outcome and main results. When necessary, an email was sent to the original authors to try to get OR or HR data that was not included in their original articles.

### Quality assessment

The same two reviewers (IJF-A and AIC-V) assessed the risk of bias of the included observational longitudinal cohort studies using the Newcastle Ottawa Scale (NOS) [38]. The NOS has been decribed as a reliable and valid tool for assessing the quality of observational longitudinal cohort studies [38, 39].

### Data synthesis and analysis

To assess the overall quality and the strength of the evidence per outcome, the Grading of Recommendations Assessment, Development and Evaluation (GRADE) approach was used [40, 41]. Two researchers (IJF-A and AIC-V) judged whether these factors were present for each outcome reported at least in two studies. Meta-analysis was conducted for each outcome reported in two or more studies, as long as studies assessed the same outcome with the same functional test and the same measurement unit, that is, HR or OR. Outcomes not included in the meta-analysis were reported using a descriptive quantitative analysis. Thus, the most relevant summary measure with the 95% Confidence Interval (95%CI) for each study was provided. The most relevant summary measure with its 95%CI was extracted of adjusted multivariate models when it was possible. In each meta-analysis it was decided to use the inverse variance as statistical method, fixed effects as analysis model and the HR or OR as effect measures. Heterogeneity was assessed using I^2^ statistic [42, 43]. Values of > 25% is considered as low heterogeneity, > 50% moderate heterogeneity, and > 75% high heterogeneity [42, 43]. When heterogeneity was moderate or high, random effects were used as analysis model. Moreover, when meta-analyses included patients with HF with reduced (HFrEF) and preserved (HFpEF) ejection fraction or meta-analyses revealed high heterogeneity, as long as the outcome was reported by three or more studies, sensitivity analyses were conducted including studies dealing only with patients with HFrEF because the inclusion of patients with different ejection fraction could be a source of heterogeneity or could bias the results. The mean effect sizes, 95% CI, and I^2^ were calculated for each outcome and used to create forest plots for visualization of each meta-analysis using the Review Manager (RevMan) version 5.3 [44].

## Results

### Characteristics of included studies

**Fig. 1:**
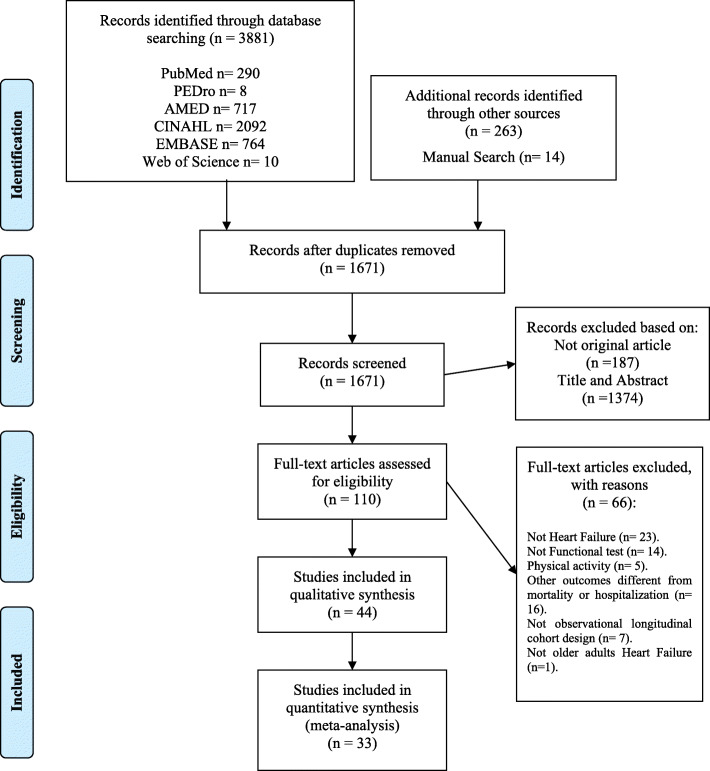
Flow-Diagram. PRISMA 2009. *From:* Moher D, Liberati A, Tetzlaff J, Altman DG, The PRISMA Group (2009). *P*referred *R*eporting *I*tems for *S*ystematic Reviews and *M*eta-*A*nalyses: The PRISMA Statement. PLoS Med 6(6): e1000097. doi:10.1371/journal.pmed1000097. For more information, visit www.prisma-statement.org

A total of 3881 citations were identified through electronic databases, with 263 additional studies identified through Grey Literature Sources and 14 studies identified through manual search. One thousand six hundred seventy-one titles and abstracts were screened and 110 original papers were assessed. The number of studies retrieved from each database and the number of studies excluded in each screening phase are shown in Fig. 1. The full reference of excluded studies in the second stage (*n* = 66) is reported in Additional file 3**.** The conflict of interest of included studies is shown in Additional file 4. Of these, 44 observational longitudinal cohort studies (prospective or retrospective) with a total of 22,598 patients with HF were included. Twenty of the included studies (45.45%) reported only patients with HFrEF. Twenty one of the included studies (47.72%) showed patients with HFrEF and HFpEF. The 6MWT was the most used test (*n* = 33) followed by the Gait Speed test (*n* = 8) and the SPPB (*n* = 4). The characteristics of the included observational longitudinal cohort studies are reported in Table 1.

**Table 1:** Characteristics of included studies

Study (first author and year)	Region	Setting	Design	Study Characteristics: Groups, Sample Size (%Male), Age	Heart Failure Diagnosis
Six Minutes Walking Test (6MWT)					
Brenyo et al. [45], 2012.	United States, Canada, and Europe	Clinical Care Setting (110 Secondary Care Centres)	Retrospective	**High Performance.** > 350 m: *n* = 1021 (82%). 62.5 ± 10.5 years. **Low Performance.** ≤ 350 m: *n* = 744 (66.4%). 66.8 ± 10.7 years.	HFrEF LVEF < 30% (29 ± 3%)
Ferreira et al. [46], 2019	11 European Countries	Clinical Care Setting (69 Secondary Care Centres)	Prospective	**High Performance.** > 360 m: *n* = 537 (86.6%). 62 ± 11.0 years. **Middle Performance.** 241-360 m: *n* = 586 (77.3%). 67 ± 12.0 years. **Low Performance.** ≤ 240 m: *n* = 591 (63%). 73 ± 10.0 years.	HFrEF LVEF = 30% (25–38%)
Wegrzynowska-Teodorczyk et al. [47], 2013.	Poland	Clinical Care Setting (Secondary Care)	Prospective	**All:** *n* = 243 (100%). 60 ± 11.0 years. **High Performance.** > 468 m. NS. **Low Performance.** ≤ 468 m. NS.	HFrEF LVEF ≤ 45% (29 ± 8%)
Bittner et al. [48], 1993.	United States, Canada, and Belgium	Clinical Care Setting (20 Tertiary Care Hospitals)	Prospective	**All:** *n* = 898 (78%). 59 ± 12.0 years. **High Performance.** ≥ 450: *n* = 201. NS. **Middle Performance.** 375–450: *n* = 215. NS. **Low Performance.** 300–375: *n* = 241. NS. **Very Low Performance.** < 300: *n* = 176. NS.	Congestive HFrEF LVEF ≤ 45%
Arslan et al. [49], 2007.	Turkey	Not Reported	Prospective	**All:** *n* = 43 (86%). 62 ± 10.0 years. **High Performance.** > 300 m. NS. **Low Performance.** ≤ 300 m. NS.	HFrEF LVEF ≤ 40% (0.35 ± 0.06%)
Lee et al. [50], 2006.	Singapore (Asian)	Clinical Care Setting (Primary and Secondary Care)	Prospective	**All:** *n* = 668 (67.4%). 66.1 ± 12.3 years. **High Performance.** > 370 m: *n* = 87. NS. **Middle Performance.** 311-370 m: *n* = 84. NS. **Low Performance.** 231-310 m: *n* = 87. NS. **Very Low Performance.** 75-230 m: *n* = 128. NS.	HFrEF LVEF < 40%
Curtis et al. [51], 2004.	United States and Canada	Clinical Care Setting (39 Secondary Care Centres)	Prospective	**High Performance.** > 400 m: *n* = 131 (91.6%). 60.0 ± 11.0 years. **Middle Performance.** 301-400 m: *n* = 210 (76.7%). 63.4 ± 10.8 years. **Low Performance.** 201-300 m: *n* = 118 (61.9%). 66.8 ± 10.4 years. **Very Low Performance.** ≤ 200 m: *n* = 82 (54.9%). 70.9 ± 12.8 years	HFrEF and HFpEF LVEF < 45% (HFrEF) LVEF > 45% (HFpEF)
Ingle et al. [52], 2014.	United Kingdom	Not Reported	Prospective	**All:** *n* = 1667 (75%). 72 (65–77) years. **High Performance.** > 360 m: *n* = NS. 64.9 ± 10.6 years. **Middle Performance.** 241-360 m: *n* = NS. 71.3 ± 8.8 years. **Low Performance.** 46-240 m: *n* = NS. 72.9 ± 9.6 years. **Very Low Performance.** ≤ 45 m: *n* = NS. 72.4 ± 10.6 years.	HFrEF LVEF < 45%
Alahdab et al. [53], 2009.	USA	Clinical Care Setting (Tertiary Care Hospital)	Prospective	**High Performance.** > 200 m: *n* = 103 (75.7%). 50.4 ± 12.2 years. **Low Performance.** ≤ 200 m: *n* = 95 (49.5%). 59.4 ± 12.2 years.	Acute Decompensated HFrEF and HFpEF LVEF ≤ 40% (HFrEF) LVEF > 40% (HFpEF)
Mangla et al. [54], 2013.	USA	Clinical Care Setting (Secondary Care)	Prospective	**All:** *n* = 900 (53%). 63.6 years. **High Performance.** > 189 m. NS. **Low Performance.** ≤ 189 m. NS.	HFpEF and HFrEF LVEF ≤ 40% (HFrEF) LVEF > 40% (HFpEF)
Hasin et al. [55], 2012.	USA	Clinical Care Setting (Secondary Care)	Retrospective	**High Performance.** ≥ 300 m: *n* = 45 (87%). 65 (53–69) years. **Low Performance.** < 300 m: *n* = 20 (75%). 68 (59–74) years.	HFrEF LVEF < 40% (20–31%)
Passantino et al. [56], 2006.	Italy	Clinical Care Setting (Secondary Care)	Prospective	**All:** n: 476 (79%). 63.6 ± 11.9 years. **High Performance.** ≥ 300 m: *n* = 301. NS. **Low Performance.** < 300 m: *n* = 175. NS.	HFrEF LVEF < 40% (29.8 ± 9.7)
Howie-Esquivel et al. [57], 2008.	USA	An Academic Medical Centre	Prospective	**High Performance.** > 200 m: *n* = 21 (73.3%). 61.7 ± 17.3 years. **Low Performance.** ≤ 200 m: *n* = 23 (26.7%). 57.6 ± 20.0 years.	Descompensated HFpEF and HFrEF LVEF < 40% (HFrEF) LVEF ≥ 40% (HFpEF)
Zotter-Tufaro et al. [58], 2015.	Austria	Not Reported	Prospective	**High Performance.** > 300 m: *n* = 72 (31.95%). 67.8 ± 9.1 years. **Low Performance.** ≤ 300 m: *n* = 70 (28.6%). 73.1 ± 7.4 years.	HFpEF LVEF ≥ 50%
Boxer et al. [59], 2010.	USA	University of Connecticut Health Centre	Prospective	**All:** *n* = 60 (71.66%). 78 ± 12.0 years. **High Performance.** > 300 m. NS. **Low Performance.** ≤ 300 m. NS.	HFrEF LVEF ≤ 40%
Ingle et al. [60], 2014	United Kingdom	Not Reported	Prospective	**All:** *n* = 600 (75%). 77.8 (71.5–83.6) years. **High Performance.** > 365 m. *n* = NS. **Middle Performance.** 271–365 m. *n* = NS. **Low Performance.** 61–270 m. *n* = NS. **Very Low Performance.** < 60 m. *n* = NS.	HFrEF LVEF < 45%
Guazzi et al. [61], 2009.	Italy	Clinical Care Setting (Secondary Care)	Prospective	**All**: *n* = 253 (78.66%). 61.9 ± 10.1 years. **High Performance.** > 300 m. *n* = 175. NS. **Low Performance.** ≤ 300 m. *n* = 78. NS	HFpEF and HFrEF LVEF < 50% (HFrEF) LVEF ≥ 50% (HFpEF)
McCabe et al. [62], 2017.	USA	An University Hospital	Prospective	**All:** *n* = 71 (57.7%). 52.6 ± 12.3 years. **High Performance.** > 300 m. NS. **Low Performance.** ≤ 300 m. NS.	HFpEF and HFrEF LVEF = 24.4 ± 13.5
Vegh et al. [63], 2014.	USA	Clinical Care Setting (Secondary Care)	Prospective	**All:** *n* = 164 (77%). 67.3 ± 12.9 years. **High Performance.** ≥ 350 m. NS. **Middle Performance.** 280-350 m. NS. **Low Performance.** < 280 m. NS.	HFrEF LVEF = 25% ± 7%.
Roul et al. [64], 1998.	France	Not Reported	Prospective	**All:** *n* = 121 (81.8%). 59 ± 11 years. **High Performance.** > 300 m. NS. **Low Performance.** ≤ 300 m. NS.	HFrEF LVEF = 29.6% ± 13%
Frankenstein et al. [65], 2008.	Germany	Specialised HF clinic at the University of Heidelberg	Prospective	**All:** *n* = 1035 (80.2%) 54.9 ± 11.5 years. **Mean 6MWT**: 459 m ± 113 m	HFrEF LVEF ≤ 40%
Mene-Afejuku et al. [66], 2017.	Nigeria	Not Reported	Prospective	**All:** *n* = 100 (NS). 64.02 ± 12.88 years. **High Performance.** 314.66 m ± 48.17 m. *n* = 59 (NS). 66.32 ± 12.29 years. **Low Performance.** 260.59 m ± 66.65 m. *n* = 41 (NS). 61.71 ± 13.46 years.	HHF (HFrEF and HFpEF) LVEF ≤ 40% (HFrEF) LVEF > 40% (HFpEF)
Ingle et al. [67], 2007	United Kingdom	Not Reported	Prospective	**All:** *n* = 1592 (60%). 74 (67–80) years. **High Performance.** ≥ 421 m. NS. **Middle Performance.** 346–420 m. NS. **Low Performance.** 241–345 m. NS. **Very Low Performance.** ≥ 240 m. NS.	HFrEF LVEF ≤ 45%
Rostagno et al. [68], 2003.	Italy	Clinical Care Setting (Secondary Care)	Prospective	**All:** *n* = 214 (93%). 53.7 (29–70) years. **High Performance.** ≥ 450 m. NS. **Middle Performance.** 300–450 m. NS. **Low Performance.** < 300 m. NS.	Congestive HFpEF and HFrEF LVEF < 50% (HFrEF) LVEF ≥ 50% (HFpEF)
Cahalin et al. [69], 1996.	USA	Clinical Care Setting (Secondary Care)	Prospective	**All:** *n* = 45 (89%). 49 ± 8 years. **High Performance.** ≥ 300 m. NS. **Low Performance.** < 300 m. NS.	HFrEF LVEF = 20 ± 6
Frankenstein et al. [70], 2008.	Germany	Specialised HF clinic at the University of Heidelberg	Prospective	**All:** *n* = 1069 (80.6%) 55.2 ± 11.7 years. **Mean 6MWT**: 456 m ± 114 m	HFrEF LVEF = 29% ± 10%
Rubim et al. [71], 2006.	Brazil	Clinical Care Setting (Secondary Care)	Prospective	**All:** *n* = 176 (67%). 58.32 ± 12.7 years. **Mean 6MWT**: 521.11 m ± 76.1 m. **High Performance.** ≥ 520 m. NS. **Low Performance.** < 520 m. NS.	HFpEF and HFrEF LVEF = 34.91% ± 12.4%
Kanagala et al. [72], 2019.	United Kingdom	Clinical Care Setting (Tertiary Care Hospital)	Prospective	**All:** *n* = 140 (49%). 73 ± 9.0 years. **Mean 6MWT**: 180 m (120 m–250 m)	HFpEF and HFrEF LVEF > 50%
Zugck et al. [73], 2001.	Germany	Medical Clinic of the University of Heidelberg	Prospective	**All:** *n* = 208 (82%). 54 ± 10 years. **Mean 6MWT**: 455 m ± 107 m (170 m–692 m)	HFrEF LVEF ≤ 40%
Cahalin et al. [74], 2013.	Italy	Clinical Care Setting (Secondary Care)	Prospective	**All:** *n* = 258 (NS). 63 ± 8.7 years. **High Performance.** > 300 m. NS. **Low Performance.** ≤ 300 m. NS.	HFpEF and HFrEF LVEF < 50% (HFrEF) LVEF ≥5 0% (HFpEF)
Reibis et al. [75], 2010.	Germany	Clinical Care Setting (Secondary Care)	Prospective	**All:** *n* = 1346 (73%). 64 ± 10 years. **Mean 6MWT**: 350.1 m ± 148.6 m	HFrEF LVEF < 45%
Castel et al. [76], 2009.	Spain	Not Reported	Retrospective	**All:** *n* = 155 (82%). 68.6 ± 7.8 years. **High Performance.** > 400 m. NS. **Middle Performance.** 310-400 m. NS. **Low Performance.** 225-310 m. NS. **Very Low Performance.** < 225 m. NS.	HFrEF LVEF ≤ 45%
Kamiya et al. [77], 2017.	Japan	Clinical Care Setting (Secondary Care Centre)	Retrospective	**All:** *n* = 1474 (68%). 72.2 ± 7.1 years. **High Performance.** ≥ 446 m: *n* = 485 (84%). 68.5 ± 5.6 years. **Middle Performance.** 342-445 m: *n* = 497 (69%). 71.5 ± 6.3 years. **Low Performance.** ≤ 341 m: *n* = 492 (52%). 76.5 ± 7.0 years.	HFpEF and HFrEF LVEF = 52.7 ± 15.4
Short Physical Performance Battery (SPPB)					
García et al. [78], 2019.	Spain	Clinical Care Setting (Secondary Care)	Prospective	**High Performance.** SPPB > 7: *n* = 37 (54.1%). 83 ± 5.7 years. **Low Performance.** SPPB≤ 7: *n* = 49 (30.6%). 86 ± 6.7 years.	Acute HF
Hornsby et al. [79], 2019.	USA	University of Michigan	Prospective	**High Performance.** SPPB≥ 10 points: *n* = 22 (55%). 64 ± 13.0 years. **Middle Performance.** SPPB = 7–9 points: *n* = 53 (42%). 67 ± 12.0 years. **Low Performance.** SPPB≤ 6 points: *n* = 39 (36%). 72 ± 13.0 years.	HFpEF HF LVEF ≥ 50%
Chiarantini et al. [80], 2010.	Italy	Clinical Care Setting (Secondary Care)	Prospective	**All:** *n* = 157 (50.3%). 80 ± 0.5 years. **High Performance.** SPPB = 9–12: *n* = 32. NS. **Middle Performance.** SPPB = 5–8: *n* = 45. NS. **Low Performance.** SPPB = 1–4: *n* = 33. NS. **Very Low Performance.** SPPB = 0: *n* = 47. NS.	Descompensated HFrEF and HFpEF LVEF < 45% (HFrEF) LVEF ≥ 45% (HFpEF)
Zaharias et al. [81], 2014.	USA	Clinical Care Setting (Secondary Care)	Prospective	**All:** *n* = 32 (78.1%). 58.2 ± 13.6 years. **High Performance.** SPPB = 10–12: *n* = 7. NS. **Middle Performance.** SPPB = 7–9: *n* = 8. NS. **Low Performance.** SPPB = 4–6: *n* = 12. NS. **Very Low Performance.** SPPB = 0–3: *n* = 4. NS.	HFrEF and HFpEF LVEF < 40% (HFrEF) LVEF ≥ 40% (HFpEF)
Gait Speed (GS)					
Lo et al. [82], 2015.	USA	Community Based Population	Prospective	**High Performance.** GS ≥ 0.8 m/s: *n* = 553 (59%). 73 ± 5.0 years. **Low Performance.** GS < 0.8 m/s: *n* = 566 (39%). 76 ± 6.0 years.	HFpEF and HFrEF LVEF < 45% (HFrEF) LVEF ≥ 45% (HFpEF)
Pulignano et al. [83], 2016.	Italy	Clinical Care Setting (7 Secondary Care Centres)	Prospective	**High Performance.** GS ≥ 1.0 m/s: *n* = 88 (64.8%). 76.4 ± 4.8 years. **Middle Performance.** GS = 0.66–0.99 m/s: *n* = 128 (60.9%). 77.1 ± 4.7 years **Low Performance.** GS ≤ 0.65 m/s: *n* = 115 (48.7%). 80.2 ± 5.6 years.	HFpEF and HFrEF LVEF < 45% (HFrEF) LVEF ≥ 45% (HFpEF)
Chaudhry et al. [84], 2013.	USA	Not Reported	Prospective	**All:** *n* = 758 (49.5%). 79.7 ± 6.2 years. **High Performance.** GS > 0.8 m/s: *n* = 441. NS. **Low Performance.** GS ≤ 0.8 m/s: *n* = 317. NS.	HFpEF and HFrEF LVEF < 45% (HFrEF) LVEF ≥ 45% (HFpEF)
Tanaka et al. [85], 2018.	Japan	Kitasato University Hospital	Retrospective	**All:** *n* = 603 (62.7%). 74.9 ± 6.2 years. **High Performance.** GS > 1.14 m/s: *n* = 154. NS. **Middle Performance.** GS = 1.0–1.14 m/s. *n* = 149. NS. **Low Performance.** GS = 0.82–0.99 m/s. *n* = 150. NS. **Very Low Performance.** GS < 0.82 m/s: *n* = 150. NS.	Acute HFpEF and HFrEF LVEF < 40% (HFrEF) LVEF ≥ 40% (HFpEF)
Tanaka et al. [86], 2019.	Japan	Kitasato University Hospital	Retrospective	**High Performance.** GS ≥ 0.8 m/s: *n* = 194 (72.7%). 73.1 ± 6.7 years. **Low Performance.** GS < 0.8 m/s: *n* = 194 (44.8%). 76.5 ± 8.4 years.	Acute HFpEF and HFrEF LVEF < 40% (HFrEF) LVEF ≥ 40% (HFpEF)
Rodríguez-Pascual et al. [87], 2017.	Spain	Clinical Care Setting (6 Secondary Care Centres)	Prospective	**High Performance.** GS ≥ 0.65 m/s: *n* = 211 (47.9%). 84.4 ± 9.4 years. **Low Performance.** GS < 0.65 m/s: *n* = 286 (32.5%). 85.7 ± 5.1 years.	HFpEF and HFrEF LVEF ≤ 45% (HFrEF) LVEF > 45% (HFpEF)
Vidán et al. [88], 2016.	Spain	Clinical Care Setting (Secondary Care Centre)	Prospective	**All:** *n* = 416 (50.5%). 80.0 ± 6.1 years. **High Performance.** GS ≥ 0.65 m/s. NS. **Low Performance.** GS < 0.65 m/s. NS.	HFpEF and HFrEF LVEF < 50% (HFrEF) LVEF ≥ 45% (HFpEF) LVEF = 43.4% ± 14.7%
Kamiya et al. [77], 2017.	Japan	Clinical Care Setting (Secondary Care Centre)	Retrospective	**All:** *n* = 1474 (68%). 72.2 ± 7.1 years. **High Performance.** GS ≥ 1.17 m/s: *n* = 489 (82%). 68.7 ± 5.5 years. **Middle Performance.** GS = 0.95–1.160 m/s: *n* = 489 (67%). 71.8 ± 6.6 years. **Low Performance.** GS ≥ 0.94 m/s: *n* = 496 (55%). 76.1 ± 7.2 years.	HFpEF and HFrEF LVEF = 52.7 ± 15.4

*m* Meters. *HF* Heart Failure. *LVEF* Left Ventricular Ejection Fraction. *NS* Not Specified. *HFrEF* Patients with Heart Failure with Reduced Ejection Fraction (Systolic Heart Failure). *HFpEF* Patients with Heart Failure with Preserved Ejection Fraction (Diastolic Heart Failure). *HHF* Hypertensive Heart Failure. *SPPB* Short Physical Performance Battery. *GS* Gait Speed

### Meta-analyses

The outcomes assessed by each study, as well as the main results, the risk of bias summary and the GRADE summary are shown in Table 2. Forest plots and effect sizes of each meta-analysis can also be seen in Additional file 5.

**Table 2:** Outcomes, Results, Risk of Bias of Included Studies and Level of Evidence per Outcome according to GRADE Criteria

Study (first author and year)	Functional Test	Follow-Up	Outcomes	Main Results	Risk of Bias	Level of Evidence (GRADE)
Brenyo et al. [45], 2012.	6MWT	4 years	**Incident HF and Mortality** ≤ 350 m VS > 350 m	HR = 1.73 95%CI [1.29–2.33]***	**Low**	**Not** **Reported**
			**Incident HF and Mortality** Per 100-m decreased	HR = 1.25 95%CI [1.09–1.44]***		
			**All-Cause Mortality** ≤ 350 m VS > 350 m	HR = 2.40 95%CI [1.42–4.08]***		**Moderate**
			**All-Cause Mortality** Per 100-m decreased	HR = 1.32 95%CI [1.05–1.66]**		
Ferreira et al. [46], 2019.	6MWT	21 months (9–26 months)	**Hospitalisation and Mortality** 241-360 m VS > 360 m	HR = 1.44 95%CI [1.14–1.80]**	**Moderate**	**Low**
			**Hospitalisation and Mortality** ≤ 240 m VS > 360 m	HR = 1.73 95%CI [1.38–2.18]***		
			**Hospitalisation and Mortality** Per each 50 m decreased	HR = 1.08 95%CI [1.04–1.11]***		
			**All-Cause Mortality** 241-360 m VS > 360 m	HR = 1.49 95%CI [1.08–2.06]**		**Moderate**
			**All-Cause Mortality** ≤ 240 m VS > 360 m	HR = 2.41 95%CI [1.76–3.29]***		
			**All-Cause Mortality** Per each 50 m decreased	HR = 1.14 95%CI [1.09–1.18]***		
Wegrzynowska-Teodorczyk et al. [47], 2013.	6MWT	1 year	**HF Mortality** ≤ 468 m VS > 468 m	HR = 3.22 95%CI [1.17–8.86]**	**Low**	**Moderate**
			**Hospitalisation and Mortality** ≤ 468 m VS > 468 m	HR = 2.77 95%CI [1.30–5.88]**		**Low**
		3 years	**HF Mortality** ≤ 468 m VS > 468 m	HR = 2.18 95%CI [1.18–4.03]**		**Moderate**
			**Hospitalisation and Mortality** ≤ 468 m VS > 468 m	HR = 1.71 95%CI [1.08–2.72]**		**Low**
Bittner et al. [48], 1993.	6MWT	1 year (242 ± 82 days)	**All-Cause Mortality** Per each 120 m decreased	OR = 1.50 95%CI [1.11–2.03]**	**Low**	**Moderate**
			**HF Hospitalisation** Per each 120 m decreased	OR = 2.60 95%CI [1.78–3.80]***		**Low**
			**Hospitalisation and Mortality** Per each 120 m decreased	OR = 1.77 95%CI [1.38–2.26]***		**Low**
			**All-Cause Mortality** < 300 m VS ≥ 450 m	OR = 3.7 95%CI [1.44–9.55]**		**Moderate**
			**All-Cause Mortality** 300-375 m VS ≥ 450 m	OR = 2.78 95%CI [1.09–7.11]**		
			**All-Cause Mortality** 375-450 m VS ≥ 450 m	OR = 1.42 95%CI [0.50–4.06]*		
			**All-Cause Hospitalisation** < 300 m VS ≥ 450 m	OR = 14.02 95%CI [4.90–40.14]***		**Low**
			**All-Cause Hospitalisation** 300-375 m VS ≥ 450 m	OR = 6.21 95%CI [2.14–18.08]***		
			**All-Cause Hospitalisation** 375-450 m VS ≥ 450 m	OR = 1.90 95%CI [0.56–6.42]*		
Arslan et al. [49], 2007.	6MWT	2 years (18 ± 6 months)	**HF Mortality** ≤ 300 m VS > 300 m	HR = 2.38 95%CI [2.02–5.76]**	**Moderate**	**Moderate**
Lee et al. [50], 2006.	6MWT	36 ± 12 months	**Hospitalisation and Mortality** 75-230 m VS > 370 m.	OR = 3.5 95%CI [1.1–11.7]**	**Low**	**Low**
			**Hospitalisation and Mortality** 231-310 m VS > 370 m	OR = 3.4 95%CI [1.01–11.5]**		
			**Hospitalisation and Mortality** 311-370 m VS > 370 m	OR = 4.9 95%CI [1.5–16.0]**		
Curtis et al. [51], 2004.	6MWT	32 months	**All-Cause Mortality** ≤ 200 m VS > 400 m	HR = 1.59 95%CI [0.88–2.86]*	**Low**	**Moderate**
			**All-Cause Mortality** 201-300 m VS > 400 m	HR = 1.01 95%CI [0.57–1.79]*		
			**All-Cause Mortality** 301-400 m VS > 400 m	HR = 1.16 95%CI [0.72–1.88]*		
			**HF Mortality** ≤ 200 m VS > 400 m	HR = 2.62 95%CI [1.02–6.74]**		**Moderate**
			**HF Mortality** 201-300 m VS > 400 m	HR = 0.93 95%CI [0.34–2.55]*		
			**HF Mortality** 301-400 m VS > 400 m	HR = 0.86 95%CI [0.35–2.09]*		
			**All-Cause Hospitalisation** ≤ 200 m VS > 400 m	HR = 1.76 95%CI [1.19–2.60]**		**Low**
			**All-Cause Hospitalisation** 201-300 m VS > 400 m	HR = 1.41 95%CI [1.01–1.99]**		
			**All-Cause Hospitalisation** 301-400 m VS > 400 m	HR = 1.09 95%CI [0.80–1.47]*		
			**HF Hospitalisation** ≤ 200 m VS > 400 m	HR = 1.84 95%CI [0.97–3.49]*		**Low**
			**HF Hospitalisation** 201-300 m VS > 400 m	HR = 1.84 95%CI [1.04–3.29]**		
			**HF Hospitalisation** 301-400 m VS > 400 m	HR = 1.45 95%CI [0.85–2.45]*		
Ingle et al. [52], 2014.	6MWT	5 years	**All-Cause Mortality** Per each 10 m increased.	HR = 0.980 95%CI [0.974–0.985]***	**Low**	**Moderate**
Alahdab et al. [53], 2009.	6MWT	40 months-Mortality	**All-Cause Mortality** ≤ 200 m VS > 200 m	HR = 2.14 95%CI [1.20–3.81]**	**Low**	**Moderate**
		40 months-Mortality	**All-Cause Mortality** Per each 1 m increased	HR = 0.998 95%CI [0.995–0.999]**		
		18 months-Hospitali- zation	**HF Hospitalisation** ≤ 200 m VS > 200 m	HR = 1.62 95%CI [1.10–2.39]**		**Low**
Mangla et al. [54], 2013.	6MWT	1080 days	**Hospitalisation and Mortality** ≤ 189 m VS > 189 m in HFpEF.	OR = 2.81 95%CI [1.24–6.40]**	**Low**	**Low**
			**Hospitalisation and Mortality** ≤ 189 m VS > 189 m in HFrEF.	OR = 1.94 95%CI [1.30–2.90]**		
Hasin et al. [55], 2012.	6MWT	Median 592 days (115–1453 days)	**All-Cause Mortality** Per 10 m walked short of 300 m	HR = 1.211 95% CI [1.108–1.322]***	**Moderate**	**Moderate**
Passantino et al. [56], 2006.	6MWT	23.9 months	**All-Cause Mortality** < 300 m VS ≥ 300 m	HR = 2.66 95%CI [1.60–4.42]***	**Low**	**Moderate**
			**All-Cause Mortality** Per each 70 m decreased	HR = 2.03 95%CI [1.29–3.18]**		
Howie-Esquivel et al. [57], 2008.	6MWT	90 days	**HF Hospitalisation** > 200 m	HR = 0.99 95%CI [0.99–1.00]*	**High**	**Low**
Zotter-Tufaro et al. [58], 2015.	6MWT	14.0 ± 10.0 months	**Hospitalisation and Mortality** > 300 m VS ≤ 300 m	HR = 0.992 95%CI [0.990–0.995]***	**Moderate**	**Low**
Boxer et al. [59], 2010.	6MWT	4 years	**All-Cause Mortality** Per each 30 m increased	HR = 0.84 95%CI [0.74–0.94]**	**Moderate**	**Moderate**
Ingle et al. [60], 2014.	6MWT	8 years	**All-Cause Mortality** Per each 10 m increased	HR = 0.988 95%CI [0.981–0.995]***	**Low**	**Moderate**
Guazzi et al. [61], 2009.	6MWT	20.4 ± 16.6 months.	**Cardiac Mortality** Per each 1 m increased	HR = 0.998 95%CI [0.995–1.001]*	**Low**	**Moderate**
McCabe et al. [62], 2017.	6MWT	30 days	**HF Hospitalisation** Per each 30 m increased	OR = 0.84 95% CI [0.71–0.99]**	**Moderate**	**Low**
Vegh et al. [63], 2014.	6MWT	3 years	**HF Hospitalisation** ≥ 350 m VS < 280 m	HR = 0.61 95% CI [0.44–0.85]**	**Moderate**	**Low**
			**Hospitalisation and Mortality** ≥ 350 m VS < 280 m	HR = 0.58 95% CI [0.43–0.80]***		**Low**
			**HF Hospitalisation** ≥ 402 m VS < 256 m	HR = 0.60 95% CI [0.44–0.82]***		**Low**
			**Hospitalisation and Mortality** ≥ 402 m VS < 256 m	HR = 0.55 95% CI [0.43–0.75]***		**Low**
Roul et al. [64], 1998.	6MWT	1000 days	**Hospitalisation and Mortality** ≤ 300 m VS > 300 m	Log rank = 6.16 **	**Moderate**	**Low**
Frankenstein et al. [65], 2008.	6MWT	52.9 ± 36.2 months	**All-Cause Mortality** Per each 1 m increased	HR = 0.996 95% CI [0.995–0.997]***	**Low**	**Moderate**
Mene-Afejuku et al. [66], 2017.	6MWT	6 months	**Hospitalisation and Mortality** 314.66 m ± 48.17 m VS 260.59 m ± 66.65 m	OR = 0.819 95% CI [0.206–3.257]*	**Moderate**	**Low**
Ingle et al. [67], 2007.	6MWT	36.6 months (28.2–45.0 months)	**All-Cause Mortality** Per each 1 m increased	HR = 0.998 95% CI [0.996–1.000]*	**Low**	**Moderate**
Rostagno et al. [68], 2003.	6MWT	34 months	**All-Cause Mortality** Per each 1 m increased	HR = 0.995 95% CI [0.993–0.997]***	**Low**	**Moderate**
Cahalin et al. [69], 1996.	6MWT	62 ± 45 weeks (1–183 weeks)	**Hospitalisation and Mortality** < 300 m VS ≥ 300 m	X^2^ = 40% vs 12% **	**Moderate**	**Low**
Frankenstein et al. [70], 2008.	6MWT	42 months (22–80 months)	**All-Cause Mortality** Per each 1 m increased	HR = 0.996 95% CI [0.995–0.997]**	**Moderate**	**Moderate**
Rubim et al. [71], 2006.	6MWT	18 months (12–24 months)	**All-Cause Mortality** ≥ 520 m VS < 520 m	OR = −0.0081 95% CI [0.0029–0.0133]***	**Low**	**Moderate**
Kanagala et al. [72], 2019.	6MWT	1429 days (1157–1657 days)	**Hospitalisation and Mortality** Per each 1 m increased	HR = 0.659 95% CI [0.465–0.934]**	**Low**	**Low**
Zugck et al. [73], 2001.	6MWT	28.3 ± 14.1 months	**All-Cause Mortality** Per each 1 m increased	HR = 0.99 95% CI [0.98–0.99]**	**Moderate**	**Moderate**
Cahalin et al. [74], 2013.	6MWT	22.8 ± 22.1 months	**Cardiac Mortality** Per each 1 m increased	HR = 0.99 95% CI [0.99–0.99]**	**Low**	**Moderate**
			**Cardiac Mortality** > 300 m VS ≤ 300 m	HR = 0.18 95% CI [0.04–0.89]**		
Reibis et al. [75], 2010.	6MWT	731 ± 215 days	**All-Cause Mortality** Per each 50 m increasd	HR = 0.93 95% CI [0.86–1.00]**	**Low**	**Moderate**
Castel et al. [76], 2009.	6MWT	24.4 ± 18.1 months	**Cardiac Mortality** < 225 m VS > 400 m	HR = 5.60 95% CI [1.23–25.30]**	**Low**	**Moderate**
			**Cardiac Mortality** 225-310 m VS > 400 m	HR = 1.28 95% CI [0.23–7.08]*		
			**Cardiac Mortality** 310-400 m VS > 400 m	HR = 4.10 95% CI [0.79–21.52]*		
Kamiya et al. [77], 2017.	6MWT	2.3 ± 1.9 years	**All-Cause Mortality** Per each 10 m increased	HR = 0.96 95% CI [0.94–0.97]***	**Low**	**Moderate**
García et al. [78], 2019.	SPPB	1 year	**HF Hospitalisation** SPPB ≤ 7 VS SPPB > 7	OR = 6.7 95%CI [1.5–30.4]**	**Moderate**	**Not Reported**
			**All-Cause Mortality** SPPB ≤ 7 VS SPPB > 7	OR = 1.2 95%CI [0.3–5.4]*		**Very Low**
			**Hospitalisation and Mortality** SPPB ≤ 7 VS SPPB > 7	OR = 3.6 95%CI [1.0–12.9]**		**Very Low**
Hornsby et al. [79], 2019.	SPPB	6 months	**Hospitalisation and Mortality** Per 1-unit change in SPPB	OR = 0.81 95%CI [0.69–0.94]**	**Moderate**	**Very Low**
			**Number of All-Cause Hospitalisations** Per 1-unit change in SPPB	IRR = 0.92 95%CI [0.86–0.97]**		**Not Reported**
			**Days Hospitalized or Dead** Per 1-unit change in SPPB	IRR = 0.85 95%CI [0.73–0.99]**		**Not Reported**
Chiarantini et al. [80], 2010.	SPPB	30 months (median 444 days)	**All-Cause Mortality** SPPB 0 VS SPPB 9–12	HR = 6.06 95%CI [2.19–16.76]***	**Moderate**	**Very Low**
			**All-Cause Mortality** SPPB 1–4 VS SPPB 9–12	HR = 4.78 95%CI [1.63–14.02]**		
			**All-Cause Mortality** SPPB 5–8 VS SPPB 9–12	HR = 1.95 95%CI [0.67–5.70]*		
Zaharias et al. [81], 2014.	SPPB	3 months	**Hospitalisation and Mortality** Per each 1 point decreased	HR = 1.042 95%CI [0.89–1.23]*	**Moderate**	**Very Low**
Lo et al. [82], 2015.	Gait Speed	10 years	**All-Cause Mortality** < 0.8 m/s VS ≥ 0.8 m/s	HR = 1.37 95%CI [1.10–1.70]**	**Low**	**Low**
Pulignano et al. [83], 2016.	Gait Speed	1 year	**All-Cause Mortality** Gait speed (tertiles)	HR = 0.620 95%CI [0.434–0.884]**	**Low**	**Low**
			**HF Hospitalisation** Gait speed (tertiles)	OR = 0.697 95%CI [0.547–0.899]**		**Low**
			**All-Cause Hospitalisation** Gait speed (tertiles)	HR = 0.741 95%CI [0.613–0.895]**		**Low**
Chaudhry et al. [84], 2013.	Gait Speed	20 years	**All-Cause Hospitalisation** ≤ 0.8 m/s VS > 0.8 m/s	HR = 1.28 95%CI [1.06–1.55]**	**Low**	**Low**
			**Hospitalisation and Mortality** ≤ 0.8 m/s VS > 0.8 m/s	HR = 1.31 95%CI [1.08–1.58]**		**Low**
Tanaka et al. [85], 2018.	Gait Speed	1.7 ± 0.5 years	**All-Cause Mortality** 1.0–1.14 m/s VS > 1.14 m/s	HR = 0.80 95%CI [0.37–1.74]*	**Moderate**	**Low**
			**All-Cause Mortality** 0.82–0.99 m/s VS > 1.14 m/s	HR = 1.46 95%CI [0.75–2.83]*		
			**All-Cause Mortality** < 0.82 m/s VS > 1.14 m/s	HR = 2.65 95%CI [1.35–5.20]**		
Tanaka et al. [86], 2019.	Gait Speed	2.1 ± 1.9 years	**All-Cause Mortality** Per each 0.1 m/s increased	HR = 0.83 95% CI [0.73–0.95]**	**Low**	**Low**
			**HF Hospitalisation** Per each 0.1 m/s increased	HR = 0.91 95% CI [0.83–0.99]**		**Low**
			**Hospitalisation and Mortality** Per each 0.1 m/s increased	HR = 0.90 95% CI [0.83–0.97]**		**Low**
Rodríguez-Pascual et al. [87], 2017.	Gait Speed	1 year	**All-Cause Mortality** GS < 0.65 m/s VS GS ≥ 0.65 m/s	HR = 1.86 95% CI [0.95–3.65]*	**Low**	**Low**
			**All-Cause Hospitalisation** GS < 0.65 m/s VS GS ≥ 0.65 m/s	HR = 1.57 95% CI [0.98–2.52]*		**Low**
Vidán et al. [88], 2016.	Gait Speed	1 year	**All-Cause Mortality** GS < 0.65 m/s VS GS ≥ 0.65 m/s	HR = 1.48 95% CI [0.95–2.32]*	**Low**	**Low**
			**All-Cause Hospitalisation** GS < 0.65 m/s VS GS ≥ 0.65 m/s	OR = 1.67 95% CI [0.98–2.85]*		**Low**
Kamiya et al. [77], 2017.	Gait Speed	2.3 ± 1.9 years	**All-Cause Mortality** Per each 0.1 m/s increased	HR = 0.87 95% CI [0.81–0.93]***	**Low**	**Low**

*6MWT* Six Minutes Walking Test. *m* Meters. *HF* Heart Failure. *HR* Hazard Ratio. *CI* Confidence Interval. OR: Odds Ratio. X^2^: Chi-square test. *HFrEF* Patients with Heart Failure with Reduced Ejection Fraction (Systolic Heart Failure). *HFpEF* Patients with Heart Failure with Preserved Ejection Fraction (Diastolic Heart Failure). *SPPB* Short Physical Performance Battery. *GS* Gait Speed. *IRR* Incidence Rate Ratio. * *p* > 0.05. ** *p* < 0.05. *** *p* < 0.001

**Fig. 2:**
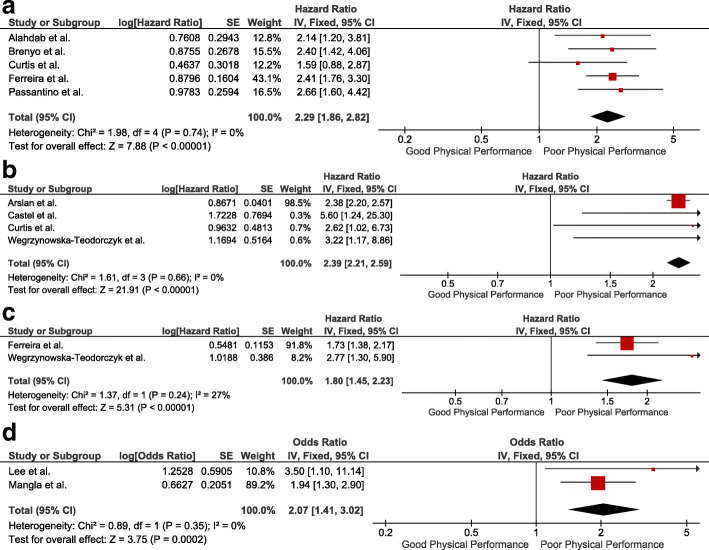
Forest Plots ilustrating the risk of All-Cause Mortality (a), the risk of HF Mortality (b) and the risk of the combined endpoint of Hospitalisation and Mortality for any cause (c and d) in the 6MWT. Patients with Poor Physical Functional Performance Versus Patients with Good Physical Functional Performance

**Fig. 3:**
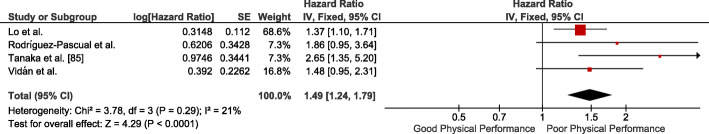
Forest Plot ilustrating the risk of All-Cause Mortality in the Gait Speed Test. Patients with slower Gait Speed Versus Patients with faster Gait Speed

Patients with HFrEF, HFpEF and acute HF who showed a poor physical functional performance in the 6MWT reported a larger risk of All-Cause of Mortality [HR = 2.29 95%CI (1.86–2.82), *p* <  0.001] than those patients who showed a good physical functional performance (Fig. 2a). Moreover, patients with HFrEF who decreased the meters (m) they walked in the 6MWT during follow-up showed larger risk of All-Cause of Mortality [HR = 1.22 95%CI (1.10–1.36), *p* <  0.001], although there was no lower risk of All-Cause of Mortality between patients with HFrEF, patients with HFpEF and patients with acute HF who increased the meters they walked in the 6MWT during follow-up (Additional file 5). Patients with HFrEF and HFpEF who showed a poor physical functional performance in the 6MWT also reported a larger risk of HF Mortality [HR = 2.39 95%CI (2.21–2.59), *p* <  0.001] than those patients who showed a good physical functional performance (Fig. 2b). Patients with HFrEF who showed a poor physical functional performance in the 6MWT also reported a larger risk of the combined endpoint of Hospitalisation and Mortality for any cause [HR = 1.80 95%CI (1.45–2.23), *p* <  0.001] or [OR = 2.07 95%CI (1.41–3.02), *p* < 0.001] than those patients who showed a good physical functional performance (Fig. 2c and Fig. 2d, respectively). Furthermore, patients with HFrEF, HFpEF and acute HF who showed a poor physical functional performance in the 6MWT reported a larger risk of HF Hospitalisation [HR = 1.68 95%CI (1.20–2.33), *p* = 0.002] than those patients who showed a good physical functional performance (Additional file 5). On the other hand, patients with HFrEF, HFpEF and acute HF who showed a slower gait speed reported a larger risk of All-Cause of Mortality [HR = 1.49 95%CI (1.24–1.79), *p* < 0.001] than those patients who showed a faster gait speed (Fig. 3), above all, when gait speed was slower than 0.65 m/s [HR = 1.59 95%CI (1.10–2.30), *p* = 0.01] (Additional file 5). Moreover, patients with HFrEF, HFpEF and acute HF who increased their gait speed during follow-up showed a lower risk of All-Cause of Mortality [HR = 0.85 95%CI (0.81–0.91) (Additional file 5). Patients with HFrEF and HFpEF who showed a slower gait speed (< 0.80 m/s) also reported a larger risk of All-Cause of Hospitalisation [HR = 1.32 95%CI (1.10–1.57), *p* = 0.002] than patients with a faster gait speed (> 0.80 m/s) (Additional file 5).

### Sensitivity analyses

The risk of All-Cause of Mortality in the 6MWT was larger when only patients with HFrEF and poor physical functional performance were assessed [HR = 2.46 95%CI (1.94–3.12), *p* < 0.001] (Additional file 6). However, the risk of HF Mortality [HR = 2.39 95%CI (2.21–2.58), *p* < 0.001] as well as the risk of All-Cause of Mortality in the 6MWT per increased units did not change when only patients with HFrEF were assessed (Additional file 6).

### Descriptive quantitative analysis

#### Physical functional performance and mortality

A score between 1 and 4 points on the SPPB was associated with a larger risk of All-Cause of Mortality (HR = 4.78 95%CI [1.63–14.02, *p* < 0.05]) in patients with HFrEF and HFpEF [80], while a score below 7 points on the SPPB was not associated with a larger risk of All-Cause of Mortality in patients with acute HF [78].

### Physical functional performance and the combined endpoint of hospitalisation and mortality

A score below 7 points on the SPPB was associated with a larger risk of the combined endpoint of hospitalisation and mortality for any cause (OR = 3.6 95%CI [1.0–12.9, *p* < 0.05]) in patients with acute HF [78]. However, per each 1-unit improved in SPPB the risk of the combined endpoint of hospitalisation and mortality for any cause could be reduced OR = 0.81 95%CI [0.69–0.94, *p* < 0.05] in patients with HFpEF [79]. Patients with HFrEF and HFpEF with a gait speed slower than 0.8 m/s also showed a larger risk of the combined endpoint of hospitalisation and mortality for any cause (HR = 1.31 95%CI [1.08–1.58, *p* < 0.05]) [84].

#### Physical functional performance and hospitalisation

Patients with HFrEF with poor physical performance in the 6MWT showed a larger risk of All-Cause of Hospitalisation [OR = 14.02 95%CI (4.90–40.14), *p* = 0.001] [48] as patients with HFrEF and HFpEF [HR = 1.41 95%CI (1.01–1.99), *p* < 0.05] [51]. A score below 7 points on the SPPB was also associated with a larger risk of HF Hospitalisation (OR = 6.7 95%CI [1.5–30.4, *p* < 0.05]) in patients with acute HF [78].

#### Risk of Bias assessment

The risk of bias of included observational longitudinal cohort studies is shown in Table 3. In summary, 26 studies (59.10%) reported a low risk of bias, and 17 studies (38,63%) showed a moderate risk of bias. Selection bias (97,72%) were usual across the included studies. Using GRADE criteria, observational longitudinal cohort studies reported a low evidence in most of the prognostic outcomes. However, HF mortality and all-cause mortality showed a moderate evidence in the 6-MWT (Table 4).

**Table 3:**
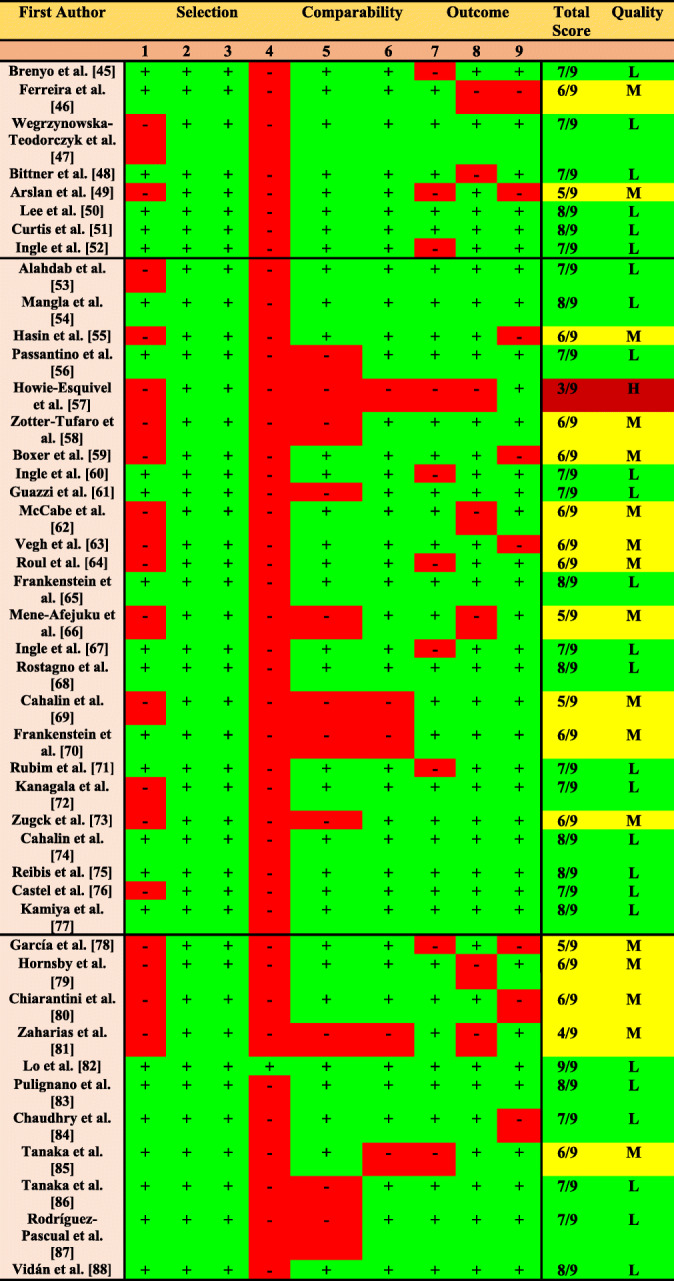
Risk of Bias Assessment of Cohort Studies (The Newcastle Ottawa Scale (NOS)).

**Note:** The NOS assigns up to a maximum of nine points for the least risk of bias based on 3 domains: selection of study groups (four points); comparability of groups (two points); and ascertainment of exposure and outcomes (three points). This checklist has been recommended for cohort studies. The risk of bias based on the NOS was classified as: Low Risk of Bias (7–9 points), Moderate Risk of Bias (4–6 points) and High Risk of Bias (0–3 points). **Abbreviations**: Quality: High Risk of Bias (H); Moderate Risk of Bias (M); Low Risk of Bias (L); NOTE. Newcastle-Ottawa Quality Assessment Scale: cohort studies: 1 = Representativeness of the exposed cohort; 2 = Selection of the non-exposed cohort; 3 = Ascertainment of exposure; 4 = Demonstration that outcome of interest was not present at start of study; 5–6 = Comparability of cohorts on the basis of the design or analysis; 7 = Assessment of outcome; 8 = Was follow-up long enough for outcomes to occur; 9 = Adequacy of follow-up of cohorts

**Table 4:** Summary of Findings and Quality of Evidence Assessment of Included Observational Longitudinal Cohort Studies (GRADE)

Summary of findings					Quality of evidence assessment (GRADE)					
Outcomes	N° studies	N° participants	Design^a^	Risk of Bias^b^	Inconsistency^c^	Indirectness ^d^	Imprecision^e^	Other ^f^	Level of Evidence	Importance
**Six Minutes Walking Test (6-MWT)**										
All-Cause Mortality	18	15,033	Observational	NO	Consistency (+ 1)	NO	NO	NO	Moderate	Critical
All-Cause Hospitalisation	2	1374	Observational	NO	Not Serious	NO	NO	NO	Low	Critical
HF Mortality	6	1493	Observational	NO	Consistency (+ 1)	NO	NO	NO	Moderate	Critical
HF Hospitalisation	6	1851	Observational	Not Serious	Not Serious	NO	Not Serious	NO	Low	Critical
Hospitalisation and Mortality	11	4788	Observational	Serious (−1)	Consistency (+ 1)	Not Serious	NO	NO	Low	Critical
**Short Physical Performance Battery (SPPB)**										
All-Cause Mortality	2	243	Observational	Very Serious (−2)	Serious (−1)	Not Serious	Serious (−1)	NO	Very Low	Critical
Hospitalisation and Mortality	3	231	Observational	Serious (−1)	Not Serious	Not Serious	Not Serious	NO	Very Low	Critical
**Gait Speed**										
All-Cause Mortality	7	4828	Observational	NO	Not Serious	NO	Not Serious	NO	Low	Critical
All-Cause Hospitalisation	4	2002	Observational	NO	Not Serious	NO	Not Serious	NO	Low	Critical
HF Hospitalisation	2	719	Observational	NO	Not Serious	NO	Not Serious	NO	Low	Critical
Hospitalisation and Mortality	2	1146	Observational	NO	Not Serious	NO	Not Serious	NO	Low	Critical

In brief, the GRADE classification was carried out according to the presence, or not, of the following identified factors: (1) study design, (2) risk of bias, (3) inconsistency of results (4) indirectness (5) imprecision, and (6) other considerations (e.g. reporting bias). The quality of the evidence based on the GRADE criteria was classified as: (1) high (further research is unlikely to change our confidence in the estimate of effect and there are no known or suspected reporting bias); (2) moderate (further research is likely to have an important effect on our confidence in the estimate of effect and could change the estimate); (3) low (further research is likely to have an important effect on our confidence in the estimate of effect and is likely to change the estimate); or (4) very low (we are uncertain about the estimate) [38]

^a^ **Design**: Observational Longitudinal Cohort Studies show a Low Level of Evidence according to GRADE

^b^ **Risk Of Bias**: > 50% (NO) of the information is from studies with low risk of bias which rarely can affect the interpretation of results. 50% (Not Serious) of the information is from studies with moderate risk of bias which could affect the interpretation of results, and 50% of the information is from studies with low risk of bias. > 50% (Serious) or > 75% (Very Serious) of the information is from studies with high/moderate risk of bias which sufficiently can affect the interpretation of results

^c^ **Inconsistency:** > 50% (Consistency) presence of high degree of consistency in the results, such as effects in same directions and not variations in the degree to which the outcome is affected (large significant effects (Hazard Ratio or Odds Ratio > 2)). > 50% (Not Serious) presence of high degree of consistency in the results, such as effects in same directions although variations in the degree to which the outcome is affected (small significant effects or large significant effects). > 50% (Serious) or > 75% (Very serious) presence of high degree of inconsistency in the results, such as effects in opposite directions, or large variations in the degree to which the outcome is affected (eg, very large and very small effects or no significant effect)

^d^ **Indirectness:** > 50% (NO) of included studies report similar population (similar HF diagnosis and similar age), as well as the same functional test (although different distances or cut-off points) and the same outcome. > 50% (Not Serious) of included studies show different HF diagnosis but population with similar age, and the same functional test (although different distances or cut-off points) and the same outcome is reported

^e^ **Imprecision:** > 50% (NO) of included studies report a 95% CI, with a narrow range (it excludes 1.0), includes large effects in the same direction and the sample size is large. > 50% (Not Serious) of included studies report a 95% CI, with a narrow range (it excludes 1.0), includes large or small effects in the same direction and the sample size could be small. > 50% (Serious) or > 75% (Very Serious) of included studies present 95% CIs with wide range (it does not exclude 1.0) and includes small effects in both directions

^f^ **Other:** Publication Bias is not suspected, and > 75% of included studies included the outcome data in a multivariate models adjusted by variables which could change the effect (NO)

## Discussion

### Main findings and comparison with other studies

The current systematic review and meta-analysis showed that patients with HFrEF and HFpEF who reported a poor physical functional performance in 6-MWT have an increased risk of all-cause of mortality and an increased risk of HF mortality. There was consistency in the risk of all-cause of mortality and HF mortality between the studies included in each meta-analysis (Fig. 2a and Fig. 2b) and the GRADE criteria also reported a moderate level of evidence per otucome. Although patients with HFrEF who decreased the meters they walked in the 6MWT during follow-up showed an increased risk of all-cause of mortality, there was no decreased risk of all-cause of mortality between patients with HFrEF and HFpEF who increased the meters they walked in the 6MWT during follow-up [52, 53, 59, 60, 65, 67, 68, 70, 73, 75, 77]. Maybe this is beacuse the most of included studies in the meta-analysis reported a decreased risk of mortality for every 1 m increased [53, 65, 67, 68, 70, 73] or every 10 m [52, 60, 77] increased, while a systematic review determined that 45 m is the clinically meaningful change in the 6MWT [89]. Patients with HF who showed a poor physical functional performance in the 6MWT also reported an increased risk of the combined endpoint of hospitalisation and mortality for any cause (Fig. 2c and Fig. 2d), an increased risk of HF hospitalisation (Additional file 5) and an increased risk of all-cause of hospitalisation [48, 51]. However, the level of evidence of those outcomes was low according to the GRADE criteria. Moreover, there was a lack of homogeneity regarding which cut-off point should be used to stratify patients with HF based on their physical functional performance in the 6MWT. A distance traveled < 300 m was the most used distance to define patients with poor physical performance in the 6MWT in this study [47, 49, 55, 56, 58, 59, 61, 62, 64, 69, 74], while a previous review reported that a distance traveled ≤350 m in 6-MWT could be the most indicative distance of poor physical functional performance and worse prognosis in patients with HF [24].

A score between 1 and 4 points on the SPPB was associated with an increased risk of all-cause of mortality in this systematic review [80]. However, in the current study a score below 7 points on the SPPB seems to be the most indicative of a worse prognosis in patients with HF since it was associated with a larger risk of the combined endpoint of hospitalisation and mortality for any cause and a larger risk of HF hospitalisation [78]. GRADE criteria showed a very low level of evidence per outcome in each outcome examined by the SPPB. Moreover, meta-analysis on physical functional performance on the SPPB and prognosis in patients with HF could not be performed. As the present review, a score below 7 points on the SPPB was also associated with large risk of all-cause mortality in older adults [90]. However, other studies reported a large risk of mortality or hospitalisation in older adults who showed a score below 5 points [80, 91–93].

Patients who showed a slower gait speed also reported an increased risk of all-cause of mortality (Fig. 3), above all, when gait speed was slower than 0.65 m/s (Additional file 5). Moreover, patients with HF who showed a slower gait speed also reported an increased risk of all-cause of hospitalisation (Additional file 5) and an increased risk of the combined endpoint of hospitalisation and mortality for any cause [84], specially when gait speed was slower than 0.80 m/s [83, 84, 86]. GRADE criteria reported a low level of evidence per outcome in each prognostic outcome in Gait Speed Test. Other studies have shown the relationship between gait speed and survival, death and hospitalisation due to HF [27, 94]. In fact, Dodson et al. [95] revealed that patients who showed a gait speed slower than 0.8 m/s were more likely to experience one-year mortality or hospitalisation than patients with gait speed faster than 0.8 m/s. Alfredsson et al. [96] also reported that patients with a gait speed slower than 0.8 m/s after a transcatheter aortic valve replacement, had 35% higher 30-day mortality than patients with faster gait speed. Chainani et al. [97] reported that gait speed and handgrip strength are associated with increased risk of cardiovascular mortality.

A meta-analysis published by Yamamoto et al. [98] reported that 6MWT were significantly associated with mortality and cardiovascular disease. Frailty has also been associated with larger risk of mortality and hospitalisation in patients with chronic HF [25, 26, 30, 31, 99]. Bagnall et al. [100] revealed that frailty patients had a risk of mortality 2- to 4-fold compared with non-frail patients after acardiac surgery or transcatheter aortic valve implantation. Gait speed is a marker of frailty, although frailty could be also assessed by the 6MWT, the SPPB or the TUG [25, 26, 30, 31, 99]. In this way, the use of functional tests seem to be useful to stratify patients with HF based on their physical functional performance and to determine their prognosis.

To our knowledge, our review is the first systematic review reporting the level of evidence per each prognostic outcome using GRADE criteria. Other reviews showed the prognostic role of the 6MWT test or the impact of the physical performance on prognosis in patients with HF, but not reported the risk of bias of included studies or the level of evidence per outcome according to GRADE criteria [22, 23, 98, 101–103].

### Implications for clinical practice

The current findings may be useful to promote functional assessments that allow stratify patients with HF according to their functional impairment. Furthermore, accurate prognostic stratification could be essential for optimizing clinical management and treatment decision making, with the aim of maintaining functionality, improving quality of life and reducing the number of hospitalisations, as well as increasing the life expectancy of patients with HF.

Adjusted medical-pharmacological treatment, in addition to improve symptoms, could prevent further cardiovascular accidents and prolong the life expectancy of patients with HF [13]. Moreover, adjusted exercise programs could reduce mortality, may improve functional capacity and quality of life, and may reduce hospitalisations [5, 8]. It has also been shown that patients with more physical activity performed weekly reported a lower risk of mortality [104–106]. Functional tests such as 6MWT, Gait Speed or SPPB may provide incremental prognostic value and could help to individualize the exercise prescription [107].

### Future research

Future research should aim to determine the optimal cut-off points for prognostic prediction and to determine the utility of functional assessments in the management and treatment of patients with HF. The following recommendations should guide future research: 1) use the same cut off point in functional tests; 2) include a large sample size with patients with HF who show different characteristics.

### Strengths and limitations of the study

The strengths of this systematic review and meta-analysis included the use of a pre-specified protocol registered on PROSPERO, the PRISMA checklist, the NOS to determine the risk of bias of each study, the GRADE criteria to assess the overall quality and the strength of the evidence per outcome, a robust search strategy complemented by a manual search, so that all studies that met the eligibility criteria could have been identified. Thus, our systematic review included 44 studies, while a previous similar review carried out by Yamamoto et al. [98] included only 22 studies.

However, there are several limitations that should be mentioned. First, the lack of uniformity among included studies, which included different cut-off points in functional tests, should be taken into account when interpreting the results. Finally, most of prognositc outcomes showed a low level of evidence per outcome according to GRADE criteria.

## Conclusion

Patients with HF who report a poor physical functional performance in the 6MWT, in the SPPB or in the Gait Speed Test, show worse prognosis than patients who report a good physical functional performance in terms of an increased risk of hospitalisation or an increased risk of mortality. However, there is a lack of homogeneity regarding which cut-off point should be used to stratify patients with HF based on their physical functional performance in the different functional tests and GRADE criteria show a low level of evidence per outcome in most of examined prognostic outcome variables.

## Supplementary information

**Additional file 1.**

**Additional file 2.**

**Additional file 3.**

**Additional file 4.**

**Additional file 5.**

**Additional file 6.**

## Data Availability

Not Applicable.

## Abbreviations

HF: Heart failure
6MWT: Six minute walking test
SPPB: Short physical performance battery
DALYs: Disability-adjusted life-years
TUG: Timed up and go test
PECOS: Participant, exposure, comparator, outcome, study design
PRISMA: Preferred reporting items for systematic reviews and meta-analyses statement
PROSPERO: International prospective register of systematic reviews
NYHA: New York heart association
OR: Odds ratio
HR: Hazard ratio
NOS: The Newcastle Ottawa scale
GRADE: Grading of recommendations assessment, development and evaluation
HFrEF: Heart failure with reduced ejection fraction
HFpEF: Heart failure with preserved ejection fraction
CI: Confidence interval
m: Meters
