# Supplementary material for: Physical functional performance and prognosis in patients with heart failure: a systematic review and meta-analysis
Source: BMC Cardiovasc Disord. 2020 Dec 9;20:512. doi: 10.1186/s12872-020-01725-5 (PMC7724724; doi:10.1186/s12872-020-01725-5)
Supplement: Supplementary file 1 — Additional file 1. [file 12872_2020_1725_MOESM1_ESM.docx]

**Appendix 2.** **Search Terms and Search Strategy**

***Search Terms***

‘heart failure’ (MeSH Terms), ‘physical functional performance’ (MeSH Terms), ‘hospitalization’ (MeSH Terms), mortality’ (MeSH Terms), ‘walking speed’ (MeSH Terms), ’Timed Up and Go’, ‘TUG’, ‘gait speed’, ‘Short Physical Performance Battery’, ‘SPPB’, ‘six minute walk test’, ‘6 minute walk test’, ‘functional performance´.

***Search Strategy***

1. PubMed, PEDro, AMED, CINAHL, EMBASE, Web of Science: (6 minute walk test) OR (six minute walk test) AND (hospitalization) AND (Heart Failure). [Title/Abstract]. 336.
2. PubMed, PEDro, AMED, CINAHL, EMBASE, Web of Science: (6 minute walk test) OR (six minute walk test) AND (mortality) AND (Heart Failure). [Title/Abstract]. 511.
3. PubMed, PEDro, AMED, CINAHL, EMBASE, Web of Science: (gait speed) OR (walking speed) AND (mortality) AND (Heart Failure). [Title/Abstract]. 995.
4. PubMed, PEDro, AMED, CINAHL, EMBASE, Web of Science: (gait speed) OR (walking speed) AND (hospitalization) AND (Heart Failure). [Title/Abstract]. 960.
5. PubMed, PEDro, AMED, CINAHL, EMBASE, Web of Science: (Timed Up and Go test) OR (TUG) AND (hospitalization) AND (Heart Failure). [Title/Abstract]. 269.
6. PubMed, PEDro, AMED, CINAHL, EMBASE, Web of Science: (Timed Up and Go test) OR (TUG) AND (mortality) AND (Heart Failure). [Title/Abstract]. 270.
7. PubMed, PEDro, AMED, CINAHL, EMBASE, Web of Science: (Short Physical Performance Battery) OR (SPPB) AND (mortality) AND (Heart Failure). [Title/Abstract]. 93.
8. PubMed, PEDro, AMED, CINAHL, EMBASE, Web of Science: (Short Physical Performance Battery) OR (SPPB) AND (hospitalization) AND (Heart Failure). [Title/Abstract]. 93.
9. PubMed, PEDro, AMED, CINAHL, EMBASE, Web of Science: (physical functional performance) OR (physical performance) AND (mortality) AND (heart failure). [Title/Abstract]. 201.
10. PubMed, PEDro, AMED, CINAHL, EMBASE, Web of Science: (physical functional performance) OR (physical performance) AND (hospitalization) AND (heart failure). [Title/Abstract]. 153.
11. PubMed Manual Search: 14.

**Grey Literature**

1. New York Academy of Medicine Grey Literature Report, Open Grey, Google Scholar: (6 minute walk test) OR (six minute walk test) AND (hospitalization) AND (Heart Failure). 7.
2. New York Academy of Medicine Grey Literature Report, Open Grey, Google Scholar: (6 minute walk test) OR (six minute walk test) AND (mortality) AND (Heart Failure). 11.
3. New York Academy of Medicine Grey Literature Report, Open Grey, Google Scholar: (gait speed) OR (walking speed) AND (mortality) AND (Heart Failure). 31.
4. New York Academy of Medicine Grey Literature Report, Open Grey, Google Scholar: (gait speed) OR (walking speed) AND (hospitalization) AND (Heart Failure). 30.
5. New York Academy of Medicine Grey Literature Report, Open Grey, Google Scholar: (Timed Up and Go test) OR (TUG) AND (hospitalization) AND (Heart Failure). 8.
6. New York Academy of Medicine Grey Literature Report, Open Grey, Google Scholar: (Timed Up and Go test) OR (TUG) AND (mortality) AND (Heart Failure). 8.
7. New York Academy of Medicine Grey Literature Report, Open Grey, Google Scholar: (Short Physical Performance Battery) OR (SPPB) AND (mortality) AND (Heart Failure). 5.
8. New York Academy of Medicine Grey Literature Report, Open Grey, Google Scholar: (Short Physical Performance Battery) OR (SPPB) AND (hospitalization) AND (Heart Failure). 5.
9. New York Academy of Medicine Grey Literature Report, Open Grey, Google Scholar: (physical functional performance) OR (physical performance) AND (mortality) AND (heart failure). 79.
10. New York Academy of Medicine Grey Literature Report, Open Grey, Google Scholar: (physical functional performance) OR (physical performance) AND (hospitalization) AND (heart failure). 79.
