# Supplementary material for: Physical functional performance and prognosis in patients with heart failure: a systematic review and meta-analysis
Source: BMC Cardiovasc Disord. 2020 Dec 9;20:512. doi: 10.1186/s12872-020-01725-5 (PMC7724724; doi:10.1186/s12872-020-01725-5)
Supplement: Supplementary file 2 — Additional file 2. [file 12872_2020_1725_MOESM2_ESM.docx]

**Appendix 3.** A short checklist for the selection of relevant studies based on inclusion and exclusion criteria.

| **Item** | **Question** | **Action** |
| --- | --- | --- |
| 1 | Was the study a original paper? | Yes, go to the next question  No, exclude |
| 2 | Did the study involve patients with Heart Failure? | Yes, go to the next question  No, exclude |
| 3 | Did the study report on physical functional performance on different functional tests such as 6MWT, TUG, SPPB or Gait Speed? | Yes, go to the next question  No, exclude |
| 4 | Did the study include at least two groups with different physical functional performance in the functional tests? | Yes, go to the next question  No, exclude |
| 5 | Did the study assess mortality or hospitalization as primary outcome? | Yes, go to the next question  No, exclude |
| 6 | Did the study include a Observational Longitudinal design? | Yes, go to the next question  No, exclude |
| 7 | Did the study provide Hazard Ratio or Odds Ratio data? | Yes, include  No, exclude |
