# Supplementary material for: Physical functional performance and prognosis in patients with heart failure: a systematic review and meta-analysis
Source: BMC Cardiovasc Disord. 2020 Dec 9;20:512. doi: 10.1186/s12872-020-01725-5 (PMC7724724; doi:10.1186/s12872-020-01725-5)
Supplement: Supplementary file 3 — Additional file 3. [file 12872_2020_1725_MOESM3_ESM.docx]

**Appendix 4**. Excluded studies in the second screening (n= 66).

Not Patients with Heart Failure. Other Cardiovascular Diseases (n= 23).

| 1 | Cipriano G, Yuri D, Bernardelli GF, Mair V, Buffolo E, Branco JNR. Analysis of 6-minute walk test safety in pre-heart transplantation patients. Arq Bras Cardiol. 2009;92(4): 294–300. |
| --- | --- |
| 2 | Georgiopoulou VV, Kalogeropoulos AP, Chowdhury R, Binongo JN B, Domingo K, Rodondi N, et al. Exercise Capacity, Heart Failure Risk, and Mortality in Older Adults: The Health ABC Study. Am J Prev Med. 2017;52(2): 144-153. |
| 3 | Miyamoto S, Nagaya N, Satoh T, Kyotani S, Sakamaki F, Fujita M NN, et al. Clinical correlates and prognostic significance of six-minute walk test in patients with primary pulmonary hypertension. Comparison with cardiopulmonary exercise testing. Am J Respir Crit Care Med. 2000;161(2 Pt 1): 487-92. |
| 4 | [Castillo-Moreno](https://pubmed.ncbi.nlm.nih.gov/?term=Castillo-Moreno+JA&cauthor_id=27567134) JA, [García-Escribano](https://pubmed.ncbi.nlm.nih.gov/?term=Garc%C3%ADa-Escribano+IA&cauthor_id=27567134) IA, [Martínez-Pascual-de-Riquelme](https://pubmed.ncbi.nlm.nih.gov/?term=Mart%C3%ADnez-Pascual-de-Riquelme+M&cauthor_id=27567134) M, [Jaulent-Huertas](https://pubmed.ncbi.nlm.nih.gov/?term=Jaulent-Huertas+L&cauthor_id=27567134) L, [Dau-Villarreal](https://pubmed.ncbi.nlm.nih.gov/?term=Dau-Villarreal+DF&cauthor_id=27567134) DF, [Rubio-Patón](https://pubmed.ncbi.nlm.nih.gov/?term=Rubio-Pat%C3%B3n+R&cauthor_id=27567134) R, et al. Prognostic Usefulness of the 6-Minute Walk Test in Patients With Severe Aortic Stenosis. Am J Cardiol. 2016;118(8): 1239–43. |
| 5 | Gurbani N, Figueira Gonçalves JM, García Bello MÁ, García-Talavera I, Afonso Díaz A. Prognostic ability of the distance-saturation product in the 6-minute walk test in patients with chronic obstructive pulmonary disease. Clin Respir J. 2019;14(4): 364-369. |
| 6 | Hsieh MH, Fang YF, Chung FT, Lee CS, Chang YC, Liu YZ, et al. Distance-saturation product of the 6-minute walk test predicts mortality of patients with non-cystic fibrosis bronchiectasis. J Thorac Dis. 2017 Sep 1;9(9): 3168–76. |
| 7 | Corsonello A, Lattanzio F, Pedone C, Garasto S, Laino I, Bustacchini S, et al. Prognostic significance of the short physical performance battery in older patients discharged from acute care hospitals. Rejuvenation Res. 2012 Feb;15(1): 41–8. |
| 8 | Flint K, Kennedy K, Arnold S V, Dodson JA, Cresci S, Alexander KP. Slow Gait Speed and Cardiac Rehabilitation Participation in Older Adults After Acute Myocardial Infarction. J Am Heart Assoc. 2018 Mar 6;7(5):1. |
| 9 | Afilalo J, [Eisenberg](https://pubmed.ncbi.nlm.nih.gov/?term=Eisenberg+MJ&cauthor_id=21050978) MJ, MorinJ-F, Bergman H, Monette J, Noiseux N, et al. Gait speed as an incremental predictor of mortality and major morbidity in elderly patients undergoing cardiac surgery. J Am Coll Cardiol. 2010 Nov 9;56(20): 1668–76. |
| 10 | Afilalo J, Sharma A, Zhang S, Brennan JM, Edwards FH, Mack MJ, et al. Gait Speed and 1-Year Mortality Following Cardiac Surgery: A Landmark Analysis From the Society of Thoracic Surgeons Adult Cardiac Surgery Database. J Am Heart Assoc. 2018 Dec 4;7(23): 1–12. |
| 11 | Kon SSC, Jones SE, Schofield SJ, Banya W, Dickson MJ, Canavan JL, et al. Gait speed and readmission following hospitalisation for acute exacerbations of COPD: a prospective study. Thorax. 2015 Dec;70(12): 1131–7. |
| 12 | Alfredsson J, Stebbins A, Brennan JM, Matsouaka R, Afilalo J, Peterson ED, et al. Gait Speed Predicts 30-Day Mortality After Transcatheter Aortic Valve Replacement: Results From the Society of Thoracic Surgeons/American College of Cardiology Transcatheter Valve Therapy Registry. Circulation. 2016 Apr 5;133(14): 1351–9. |
| 13 | Cheung C-L, Lam KSL, Cheung BMY, Cheung C-L. Evaluation of Cutpoints for Low Lean Mass and Slow Gait Speed in Predicting Death in the National Health and Nutrition Examination Survey 1999-2004. Journals Gerontol Ser A Biol Sci Med Sci. 2016 Jan;71(1): 90–5. |
| 14 | Volpato S, Cavalieri M, Sioulis F, Guerra G, Maraldi C, Zuliani G, et al. Predictive value of the Short Physical Performance Battery following hospitalization in older patients. Journals Gerontol Ser A Biol Sci Med Sci. 2011 Jan;66A(1): 89–96. |
| 15 | Matsuzawa Y, Konishi M, Akiyama E, Suzuki H, Nakayama N, Kiyokuni M, et al. Association between gait speed as a measure of frailty and risk of cardiovascular events after myocardial infarction. J Am Coll Cardiol. 2013 May 14;61(19): 1964–72. |
| 16 | Andrianopoulos V, Wouters EF, Pinto-Plata VM, [Vanfleteren](https://pubmed.ncbi.nlm.nih.gov/?term=Vanfleteren+LE&cauthor_id=26143282) LE , [Bakke](https://pubmed.ncbi.nlm.nih.gov/?term=Bakke+PS&cauthor_id=26143282) PS, [Franssen](https://pubmed.ncbi.nlm.nih.gov/?term=Franssen+FM&cauthor_id=26143282) FM, et al. Prognostic value of variables derived from the six-minute walk test in patients with COPD: Results from the ECLIPSE study. Respir Med. 2015 Sep 1;109(9): 1138–46. |
| 17 | Martin C, Chapron J, Hubert D, Kanaan R, Honoré I, Paillasseur JL, et al. Prognostic value of six minute walk test in cystic fibrosis adults. Respir Med. 2013 Dec 1;107(12): 1881–7. |
| 18 | Paciocco G, Martinez FJ, Bossone E, Pielsticker E, Gillespie B, Rubenfire M. Oxygen desaturation on the six-minute walk test and mortality in untreated primary pulmonary hypertension. Eur Respir J. 2001 Apr 1;17(4): 647–52. |
| 19 | [Polkey](https://pubmed.ncbi.nlm.nih.gov/?term=Polkey+MI&cauthor_id=23262518) MI, [Spruit](https://pubmed.ncbi.nlm.nih.gov/?term=Spruit+MA&cauthor_id=23262518) MA, [Edwards](https://pubmed.ncbi.nlm.nih.gov/?term=Edwards+LD&cauthor_id=23262518) LD, [Watkins](https://pubmed.ncbi.nlm.nih.gov/?term=Watkins+ML&cauthor_id=23262518) ML, Pinto-Plata V, Vestbo J, et al. Six-minute-walk test in chronic obstructive pulmonary disease: Minimal clinically important difference for death or hospitalization. Am J Respir Crit Care Med. 2013 Feb 15;187(4): 382–6 |
| 20 | [Beatty](https://pubmed.ncbi.nlm.nih.gov/?term=Beatty+AL&cauthor_id=22710902) AL, [Schiller](https://pubmed.ncbi.nlm.nih.gov/?term=Schiller+NB&cauthor_id=22710902) NB, [Whooley](https://pubmed.ncbi.nlm.nih.gov/?term=Whooley+MA&cauthor_id=22710902) MA. Six-minute walk test as a prognostic tool in stable coronary heart disease. Arch Intern Med. 2012 Jul 23;172(14): 1096–102. |
| 21 | Dodson JA, Arnold S V, Gosch KL, Gill TM, Spertus JA, Krumholz HM, et al. Slow Gait Speed and Risk of Mortality or Hospital Readmission After Myocardial Infarction in the Translational Research Investigating Underlying Disparities in Recovery from Acute Myocardial Infarction: Patients’ Health Status Registry. J Am Geriatr Soc. 2016 Mar;64(3): 596–601. |
| 22 | Donoghue OA, Jansen S, Dooley C, De Rooij S, Van Der Velde N, Kenny RA. Atrial fibrillation is associated with impaired mobility in community-dwelling older adults. J Am Med Dir Assoc. 2014 Dec;15(12): 929–33. |
| 23 | Sanchez CE, Hermiller JB, Pinto DS, Chetcuti SJ, Arshi A, Forrest JK, et al. Predictors and risk calculator of early unplanned hospital readmission following contemporary self-expanding transcatheter aortic valve replacement from the STS/ACC TVT-registry. Cardiovasc Revasc Med. 2019. |

Not Functional Tests (n= 14).

| 1 | Uemura Y, Shibata R, Takemoto K, Koyasu M, Ishikawa S, Murohara T W, et al. Prognostic Impact of the Preservation of Activities of Daily Living on Post- Discharge Outcomes in Patients With Acute Heart Failure. Circ J.  2018 Oct 25;82(11): 2793-2799. | | |
| --- | --- | --- | --- |
| 2 | Ellery S, Pakrashi T, Paul V, Sack S. Predicting mortality and rehospitalization in heart failure patients with home monitoring. The Home CARE pilot study.  Clin Res Cardiol. 2006;95 Suppl 3: 29-35. | | |
| 3 | Ozierański K, Balsam P, Tymińska A, Peller M, Kapłon-Cieślicka A M, Drożdż J, et al. Heart failure in elderly patients: differences in clinical characteristics and predictors of 1-year outcome in the Polish ESC-HF Long-Term Registry. Pol Arch Med Wewn. 2016 Aug 11; 126 (7-8): 502-13. | | |
| 4 | Parikh KS, Greiner MA, Suzuki T, DeVore AD, Blackshear C, Maher JF C, et al. Resting Heart Rate and Long-term Outcomes Among the African American Population: Insights From the Jackson Heart Study. JAMA Cardiol. 2017 Feb 1; 2(2): 172-180. | |  |
| 5 | Kinugasa Y, Kato M, Sugihara S, Hirai M, Yamada K, Yanagihara K Y, et al. Geriatric nutritional risk index predicts functional dependency and mortality in patients with heart failure with preserved ejection fraction. Circ J. 2013;77(3): 705-11. | |  |
| 6 | Carroll AJ, Howrey HL, Payvar S, Deshida-Such K, Kansal M BC. A Heart Failure Management Program Using Shared Medical Appointments. Version 2. Fed Pract.  Apr 2017; 34(4): 14-19. | |  |
| 7 | Cooper LB, Hammill BG, Allen LA, Lindenfeld J, Mentz RJ, Rogers JG M, et al. Assessing Frailty in Patients Undergoing Destination Therapy Left Ventricular Assist Device: Observations from Interagency Registry for Mechanically Assisted Circulatory Support. ASAIO J. Jan/Feb 2018;64 (1): 16-23. |  |  |
| 8 | Jha SR, Hannu MK, Chang S, Montgomery E, Harkess M, Wilhelm K HC, et al. The Prevalence and Prognostic Significance of Frailty in Patients With Advanced Heart Failure Referred for Heart Transplantation. Transplantation. Feb 2016;100 (2): 429-36. |  |  |
| 9 | Monika Szymik, Bożena Szyguła-Jurkiewicz, Robert Partyka, Aleksander Owczarek, Beata Spinczyk, Jerzy Chudek LP. Three-year survival of patients with chronic systolic heart failure due to hypertension: Analysis of prognostic factors. Pol Arch Med Wewn. 2012;122(11): 543–50. |  |  |
| 10 | [Madan](https://pubmed.ncbi.nlm.nih.gov/?term=Madan+SA&cauthor_id=26883168) SA, [Fida](https://pubmed.ncbi.nlm.nih.gov/?term=Fida+N&cauthor_id=26883168) N, [Barman](https://pubmed.ncbi.nlm.nih.gov/?term=Barman+P&cauthor_id=26883168) P, [Sims](https://pubmed.ncbi.nlm.nih.gov/?term=Sims+D&cauthor_id=26883168) D, [Shin](https://pubmed.ncbi.nlm.nih.gov/?term=Shin+J&cauthor_id=26883168) J, [Verghese](https://pubmed.ncbi.nlm.nih.gov/?term=Verghese+J&cauthor_id=26883168) J, et al. Frailty Assessment in Advanced Heart Failure. J Card Fail. 2016;22(10): 840–4. |  |  |
| 11 | [Saitoh](https://pubmed.ncbi.nlm.nih.gov/?term=Saitoh+M&cauthor_id=27853883) M, [Dos Santos](https://pubmed.ncbi.nlm.nih.gov/?term=Dos+Santos+MR&cauthor_id=27853883) MR, [Ebner](https://pubmed.ncbi.nlm.nih.gov/?term=Ebner+N&cauthor_id=27853883) N, [Emami](https://pubmed.ncbi.nlm.nih.gov/?term=Emami+A&cauthor_id=27853883) A, [Konishi](https://pubmed.ncbi.nlm.nih.gov/?term=Konishi+M&cauthor_id=27853883) M, [Ishida](https://pubmed.ncbi.nlm.nih.gov/?term=Ishida+J&cauthor_id=27853883) J, et al. Nutritional status and its effects on muscle wasting in patients with chronic heart failure: insights from Studies Investigating Comorbidities Aggravating Heart Failure. Wien Klin Wochenschr. 2016;128: 497–504. |  |  |
| 12 | [Sterliński](https://pubmed.ncbi.nlm.nih.gov/?term=Sterli%C5%84ski+M&cauthor_id=18058579) M, [Maciag](https://pubmed.ncbi.nlm.nih.gov/?term=Maciag+A&cauthor_id=18058579) A, [Kowalski](https://pubmed.ncbi.nlm.nih.gov/?term=Kowalski+O&cauthor_id=18058579) O, [Gościńska-Bis](https://pubmed.ncbi.nlm.nih.gov/?term=Go%C5%9Bci%C5%84ska-Bis+K&cauthor_id=18058579) K, [Pytkowski](https://pubmed.ncbi.nlm.nih.gov/?term=Pytkowski+M&cauthor_id=18058579) M, [Kowalik](https://pubmed.ncbi.nlm.nih.gov/?term=Kowalik+I&cauthor_id=18058579) I, et al. Mortality in patients with heart failure treated with cardiac resynchronisation therapy. A long-term multi-centre follow-up study. Kardiol Pol. 2007;65(11): 1287–93. |  |  |
| 13 | Perna ER, Macin SM, Cimbaro Canella JP, Augier N, Riera Stival JL, Cialzeta JR, et al. Ongoing myocardial injury in stable severe heart failure: Value of cardiac troponin T monitoring for high-risk patient identification. Circulation. 2004 Oct 19;110(16): 2376–82. |  |  |
| 14 | Saevereid HA, Schnohr P, Prescott E. Speed and duration of walking and other leisure time physical activity and the risk of heart failure: A prospective cohort study from the Copenhagen City Heart Study. PLoS One. 2014 Mar 12;9(3). |  |  |

Physical Activity and Mortality or Hospitalization (n= 5).

| 1 | Waring T, Gross K, Soucier R, ZuWallack R. Measured Physical Activity and 30-Day Rehospitalization in Heart Failure Patients. J Cardiopulm Rehabil Prev. 2017;37(2): 124–9. |
| --- | --- |
| 2 | Melin M, Hagerman I, Gonon A, Gustafsson T, Rullman E. Variability in Physical Activity Assessed with Accelerometer Is an Independent Predictor of Mortality in CHF Patients. PLoS One. 2016;11(4): e0153036. |
| 3 | Izawa KP, Watanabe S, Oka K, Hiraki K, Morio Y, Kasahara Y, et al. Usefulness of step counts to predict mortality in Japanese patients with heart failure. Am J Cardiol. 2013;111(12): 1767–71. |
| 4 | Snipelisky D, Kelly J, Levine JA, Koepp GA, Anstrom KJ, McNulty SE, et al. Accelerometer-Measured Daily Activity in Heart Failure with Preserved Ejection Fraction: Clinical Correlates and Association with Standard Heart Failure Severity Indices. Circ Hear Fail. 2017;10(6). |
| 5 | Prescher S, Schoebel C, Koehler K, Deckwart O, Wellge B, Honold M, et al. Prognostic value of serial six-minute walk tests using tele-accelerometry in patients with chronic heart failure: A pre-specified sub-study of the TIM-HF-Trial. Eur J Prev Cardiol. 2016;23(2_suppl): 21–6. |

Not Observational Longitudinal Design (n= 7).

| 1 | Giannitsi S, Bougiakli M, Bechlioulis A, Kotsia A, Michalis LK, Naka KK. 6-minute walking test: a useful tool in the management of heart failure patients. Therapeutic advances in cardiovascular disease. [Ther Adv Cardiovasc Dis](https://www.ncbi.nlm.nih.gov/pmc/journals/3336/). 2019; 13. |
| --- | --- |
| 2 | [Tallaj](https://pubmed.ncbi.nlm.nih.gov/?term=Tallaj+JA&cauthor_id=11508182) JA, [Sanderson](https://pubmed.ncbi.nlm.nih.gov/?term=Sanderson+B&cauthor_id=11508182) B, [Breland](https://pubmed.ncbi.nlm.nih.gov/?term=Breland+J&cauthor_id=11508182) J, [Adams](https://pubmed.ncbi.nlm.nih.gov/?term=Adams+C&cauthor_id=11508182) C, [Schumann](https://pubmed.ncbi.nlm.nih.gov/?term=Schumann+C&cauthor_id=11508182) C, [Bittner](https://pubmed.ncbi.nlm.nih.gov/?term=Bittner+V&cauthor_id=11508182) V. Assessment of functional outcomes using the 6-minute walk test in cardiac rehabilitation: Comparison of patients with and without left ventricular dysfunction. J Cardiopulm Rehabil. 2001;21(4): 221–4. |
| 3 | Kuschyk J, Nägele H, Heinz-Kuck K, Butter C, Lawo T, Wietholt D RS, et al. Cardiac contractility modulation treatment in patients with symptomatic heart failure despite optimal medical therapy and cardiac resynchronization therapy (CRT). Int J Cardiol.  2019 Feb 15;277: 173-177. |
| 4 | [Papathanasiou](https://pubmed.ncbi.nlm.nih.gov/?term=Papathanasiou+JV&cauthor_id=23557612) JV, [Ilieva](https://pubmed.ncbi.nlm.nih.gov/?term=Ilieva+E&cauthor_id=23557612) E, [Marinov](https://pubmed.ncbi.nlm.nih.gov/?term=Marinov+B&cauthor_id=23557612) B. Six-minute Walk Test: An Effective and Necessary Tool in Modern Cardiac Rehabilitation. Hellenic J Cardiol. Mar-Apr 2013;54(2): 126-30. |
| 5 | Woo MA, Moser DK, Stevenson LW, Stevenson WG. Six-minute walk test and heart rate variability: lack of association in advanced stages of heart failure. Am J Crit Care. 1997;6(5): 348–54 |
| 6 | Zielińska D, Bellwon J, Rynkiewicz A, Elkady MA. Prognostic value of the six-minute walk test in heart failure patients undergoing cardiac surgery: a literature review. Rehabil Res Pr. 2013;1. |
| 7 | Jaarsma T, Klompstra L, Ben Gal T, Boyne J, Vellone E, Bäck M DK, et al. Increasing exercise capacity and quality of life of patients with heart failure through Wii gaming: the rationale, design and methodology of the HF-Wii study; a multicentre randomized controlled trial. Eur J Heart Fail.  Jul 2015; 17(7): 743-8. |

Not Older Adults Heart Failure (n= 1).

| 1 | den Boer SL, Flipse DHK, van der Meulen MH, Backx APCM, du Marchie Sarvaas GJ, Ten Harkel ADJ, et al. Six-Minute Walk Test as a Predictor for Outcome in Children with Dilated Cardiomyopathy and Chronic Stable Heart Failure. Pediatr Cardiol. 2017 Mar 1;38(3): 465–71. |
| --- | --- |

Other Outcomes different from Mortality or Hospitalization (n= 16).

| 1 | Witham MD, Argo IS, Johnston DW, Struthers AD, McMurdo MET. Predictors of exercise capacity and everyday activity in older heart failure patients. Eur J Heart Fail. 2006 Mar;8(2):203–7. |
| --- | --- |
| 2 | Kono Y, Izawa H, Aoyagi Y, Ishikawa A, Sugiura T, Mori E, et al. The difference in determinant factor of six-minute walking distance between sarcopenic and non-sarcopenic elderly patients with heart failure. J Cardiol. 2020;75(1): 42–6. |
| 3 | Shoemaker MJ, Curtis AB, Vangsnes E, Dickinson MG. Clinically Meaningful Change Estimates for the Six-Minute Walk Test and Daily Activity in Individuals With Chronic Heart Failure. Cardiopulm Phys Ther J. 2013 Sep;24(3): 21–9. |
| 4 | Kurose S, Miyauchi T, Yamashita R, Tamaki S, Imai M, Nakashima Y, et al. Association of locomotive activity with sleep latency and cognitive function of elderly patients with cardiovascular disease in the maintenance phase of cardiac rehabilitation. J Cardiol. 2019 Jun 1;73(6): 530–5. |
| 5 | Hajjar I, Yang F, Sorond F, Jones RN, Milberg W, Cupples LA, et al. A novel aging phenotype of slow gait, impaired executive function, and depressive symptoms: relationship to blood pressure and other cardiovascular risks. J Gerontol A Biol Sci Med Sci. 2009;64(9): 994–1001. |
| 6 | Daullxhiu I, Haliti E, Poniku A, Ahmeti A, Hyseni V, Olloni R, et al. Predictors of exercise capacity in patients with chronic heart failure. J Cardiovasc Med. 2011 Mar;12(3): 223–5. |
| 7 | Warraich HJ, Kitzman DW, Whellan DJ, Duncan PW, Mentz RJ, Pastva AM, et al. Physical Function, Frailty, Cognition, Depression, and Quality of Life in Hospitalized Adults ≥60 Years With Acute Decompensated Heart Failure With Preserved Versus Reduced Ejection Fraction. Circ Heart Fail. 2018;11(11): e005254. |
| 8 | Floegel TA, Dickinson JM, DerAnanian C, McCarthy M, Hooker SP, Buman MP. Association of Posture and Ambulation With Function 30 Days After Hospital Discharge in Older Adults with Heart Failure. J Card Fail. 2018;24(2): 126–30. |
| 9 | Guimarães GV, Carvalho VO, Bocchi EA. Reproducibility of the self-controlled six-minute walking test in heart failure patients. Clinics (Sao Paulo). 2008;63(2): 201–6. |
| 10 | Adedoyin RA, Adeyanju SA, Balogun MO, Adebayo RA, Akintomide AO, Akinwusi PO. Prediction of functional capacity during six-minute walk among patients with chronic heart failure. Niger J Clin Pract. 2010;13(4): 379–81. |
| 11 | Zdrenghea D, Beudean M, Pop D, Zdrenghea V. Four hundred meters walking test in the evaluation of heart failure patients. Rom J Intern Med. 2010;48(1): 33–8. |
| 12 | Jehn M, Schmidt-Trucksäss A, Schuster T, Weis M, Hanssen H, Halle M, et al. Daily walking performance as an independent predictor of advanced heart failure: Prediction of exercise capacity in chronic heart failure. Am Heart J. 2009;157(2): 292–8. |
| 13 | Collins S, Thorn M, Nowak R, Levy P, Fermann G, Hiestand B, et al. Feasibility of Serial 6-min Walk Tests in Patients with Acute Heart Failure. J Clin Med. 2017;6(9): 84. |
| 14 | Costa S de A, Rassi S, Freitas EM da M, Gutierrez N da S, Boaventura FM, Sampaio LP da C, et al. Fatores Prognósticos na Insuficiência Cardíaca Grave de Etiologia Chagásica. Arq Bras Cardiol. 2017 Mar 1;108(3): 246–54 |
| 15 | Boxer RS, Wang Z, Walsh SJ, Hager D, Kenny AM. The utility of the 6-minute walk test as a measure of frailty in older adults with heart failure. Am J Geriatr Cardiol. 2008 Jan;17(1): 7–12. |
| 16 | Chaudhry SI, McAvay G, Ning Y, Allore HG, Newman AB, Gill TM. Risk factors for onset of disability among older persons newly diagnosed with heart failure: the Cardiovascular Health Study. J Card Fail. 2011;17(9): 764–70. |
