# Supplementary material for: Physical functional performance and prognosis in patients with heart failure: a systematic review and meta-analysis
Source: BMC Cardiovasc Disord. 2020 Dec 9;20:512. doi: 10.1186/s12872-020-01725-5 (PMC7724724; doi:10.1186/s12872-020-01725-5)
Supplement: Supplementary file 4 — Additional file 4. [file 12872_2020_1725_MOESM4_ESM.docx]

**Appendix 5.** Conflict of interest of included studies.

| **Fist Author and Year** | **Conflict of Interest** |
| --- | --- |
| Alahdab et. [45] | Not reported. |
| Arslan et al. [58] | Not reported. |
| Bittner et al. [43] | Not reported. |
| Boxer et al. [56] | Not reported. |
| Brenyo et al. [51] | Not reported. |
| Chaudhry et al. [50] | Not reported. |
| Chiarantini et al. [59] | Not reported. |
| Curtis et al. [44] | Not reported. |
| Ferreira et al. [52] | None declared. |
| García et al. [47] | The authors declared that they did not have conflicts of interest in relation to the article. |
| Hasin et al. [54] | Not reported. |
| Hornsby et al. [65] | Not reported. |
| Howie-Esquivel et al. [46] | Not reported. |
| Ingle et al. [53] | The authors declared that there were no conflict of interests  regarding the publication of the paper. |
| Lee et al. [62] | Not reported. |
| Lo et al. [60] | The authors had no conflicts of interest to disclose. |
| Mangla et al. [63] | The authors had no conflicts of interest to disclose. |
| Passantino et al. [55] | Not reported. |
| Pulignano et al. [48] | The authors reported that they had no relationships relevant to the contents of the paper to disclose. |
| Tanaka et al. [61] | None declared. |
| Tanaka et al. [49] | None declared. |
| Wegrzynowska-Teodorczyk et al. [57] | No author had any conflict of interest related to the data and ideas presented in the manuscript. |
| Zotter-Tufaro et al. [64] | All authors reported that they had no relationships relevant to the contents of the paper to disclose. |
| Rodríguez-Pascual et al. [66] | The authors reported no relationships that could be construed as a conflict of interest. |
| Ingle et al. [67] | The authors declared that they had no conflicts of interest concerning this article. |
| Guazzi et al. [68] | None. |
| McCabe et al. [69] | Not reported. |
| Vegh et al. [70] | Not reported. |
| Vidán et al. [71] | None declared. |
| Roul et al. [72] | Not reported. |
| Frankenstein et al. [73] | Not reported. |
| Mene-Afejuku et al. [74] | The authors reported no conflicts of interest in the work. |
| Ingle et al. [75] | None declared. |
| Rostagno et al. [76] | Not reported. |
| Cahalin et al. [77] | Not reported. |
| Frankenstein et al. [78] | Not reported. |
| Rubim et al. [79] | Not reported. |
| Kanagala et al. [80] | Lei Zhao and Jing Yang were employees of Bristol Myers Squibb which facilitated plasma NTpro-ANP analysis. All other authors declared that they had no competing interests relevant to the study. All authors also stated that they had full control of all primary data and that they agreed to allow the journal to review their data if requested. |
| Zugck et al. [81] | Not reported. |
| Cahalin et al. [82] | None declared. |
| Reibis et al. [83] | The authors declared that they had no financial or personal  relations to other parties whose interests could have affected  the content of the article in any way, either positively or negatively. |
| Castel et al. [84] | None declared. |
| Zaharias et al. [85] | The authors declared that there was no conflict of interests  regarding the publication of the paper. |
| Kamiya et al. [86] | The authors declared no potential conflicts of interest with  respect to the research, authorship, and/or publication of the  article. |
