# Supplementary material for: Physical functional performance and prognosis in patients with heart failure: a systematic review and meta-analysis
Source: BMC Cardiovasc Disord. 2020 Dec 9;20:512. doi: 10.1186/s12872-020-01725-5 (PMC7724724; doi:10.1186/s12872-020-01725-5)
Supplement: Supplementary file 5 — Additional file 5. [file 12872_2020_1725_MOESM5_ESM.docx]

**Additional file 5. Forest plots and effects sizes of each meta-analysis.**


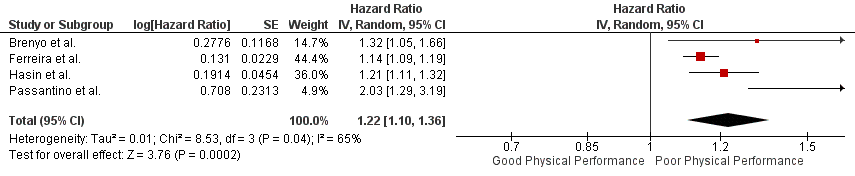


**Figure 1.** Forest Plot ilustrating the risk of All-Cause of Mortality in the 6MWT per decreased Units.


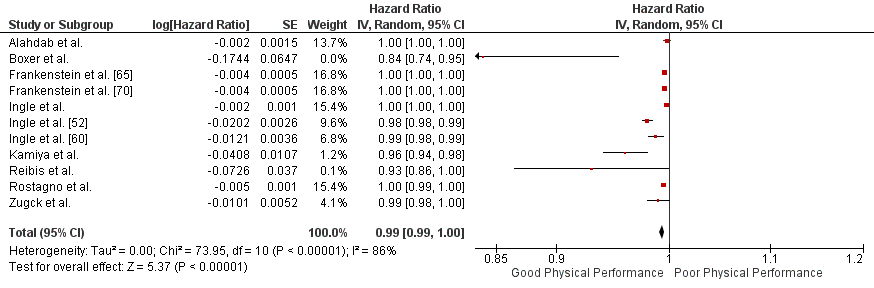


**Figure 2.** Forest Plot ilustrating the risk of All-Cause of Mortality in the 6MWT per increased Units.


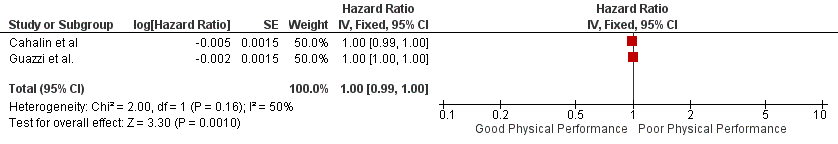


**Figure 3.** Forest Plot ilustrating the risk of HF Mortality in the 6MWT per increased Units.


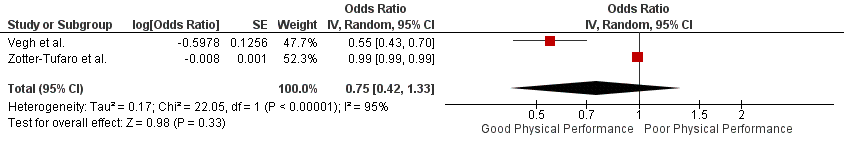


**Figure 4.** Forest Plot ilustrating the risk of the combined endpoint of Hospitalisation and Mortality for any cause in the 6MWT. Patients with Good Physical Functional Performance Versus Patients with Poor Physical Functional Performance.


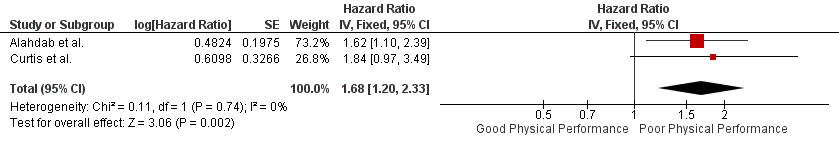


**Figure 5.** Forest Plot ilustrating the risk of HF Hospitalisation in the 6MWT. Patients with Poor Physical Functional Performance Versus Patients with Good Physical Functional Performance.


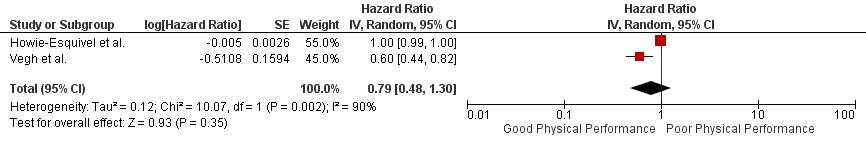


**Figure 6.** Forest Plot ilustrating the risk of HF Hospitalisation in the 6MWT. Patients with Good Physical Functional Performance Versus Patients with Poor Physical Functional Performance.


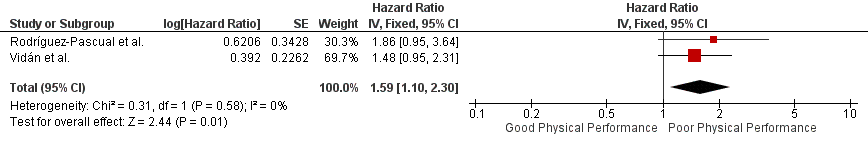


**Figure 7.** Forest Plot ilustrating the risk of All-Cause Mortality in the Gait Speed Test. Patients with slower Gait Speed (<0.65m/s) Versus Patients with faster Gait Speed (>0.65m/s).


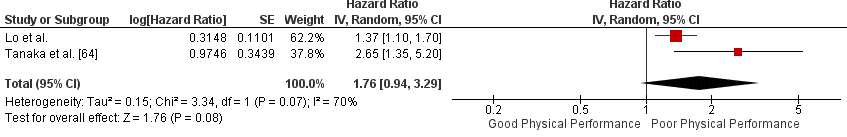


**Figure 8.** Forest Plot ilustrating the risk of All-Cause Mortality in the Gait Speed Test. Patients with slower Gait Speed (<0.80m/s) Versus Patients with faster Gait Speed (>0.80m/s).


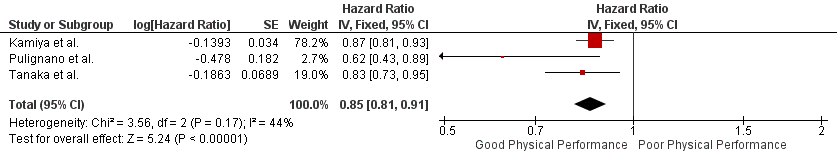


**Figure 9.** Forest Plot ilustrating the risk of All-Cause Mortality in the Gait Speed Test per increased units.

**
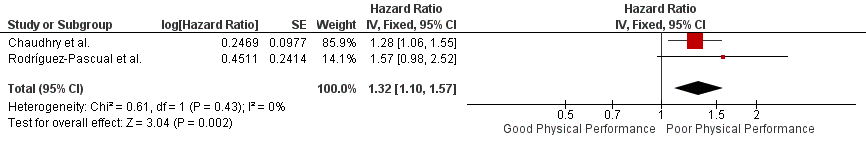
**

**Figure 10.** Forest Plot ilustrating the risk of All-Cause Hospitalisation in the Gait Speed Test. Patients with slower Gait Speed (<0.80m/s) Versus Patients with faster Gait Speed (>0.80m/s).


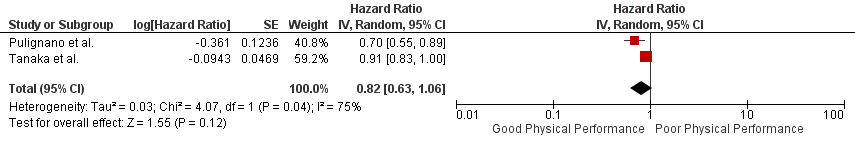


**Figure 11.** Forest Plot ilustrating the risk of HF Hospitalisation in the Gait Speed Test per increased units.
