# Supplementary material for: Physical functional performance and prognosis in patients with heart failure: a systematic review and meta-analysis
Source: BMC Cardiovasc Disord. 2020 Dec 9;20:512. doi: 10.1186/s12872-020-01725-5 (PMC7724724; doi:10.1186/s12872-020-01725-5)
Supplement: Supplementary file 6 — Additional file 6. [file 12872_2020_1725_MOESM6_ESM.docx]

**Additional file 6. Sensitivity Analyses. Forest plots and effects sizes of each meta-analysis.**


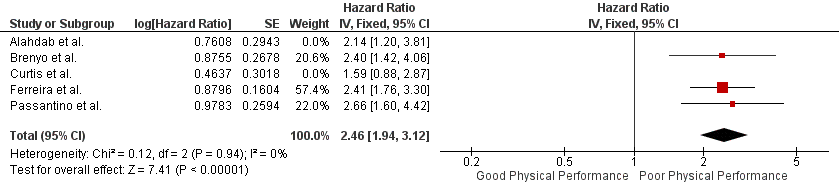


**Figure 1.** Forest Plots ilustrating the risk of All-Cause Mortality in the 6MWT. Patients with Poor Physical Functional Performance Versus Patients with Good Physical Functional Performance.


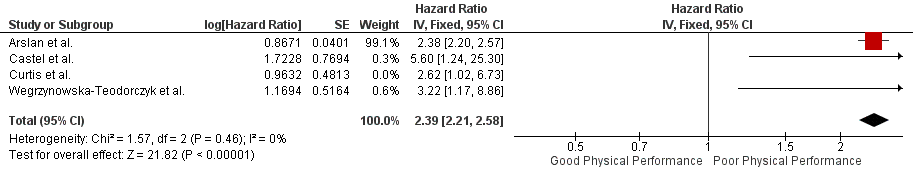


**Figure 2.** Forest Plots ilustrating the risk of HF Mortality in the 6MWT. Patients with Poor Physical Functional Performance Versus Patients with Good Physical Functional Performance.


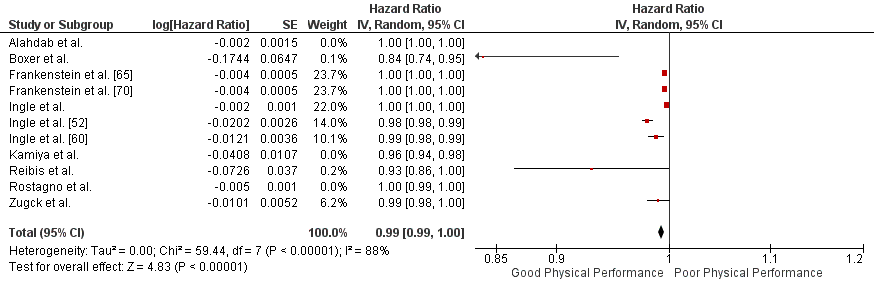


**Figure 3.** Forest Plot ilustrating the risk of All-Cause of Mortality in the 6MWT per increased Units.
